# Single-cell transcriptomics reveals cellular heterogeneity and macrophage-to-mesenchymal transition in bicuspid calcific aortic valve disease

**DOI:** 10.1186/s13062-023-00390-w

**Published:** 2023-06-30

**Authors:** Tao Lyu, Yang Liu, Binglin Li, Ran Xu, Jianghong Guo, Dan Zhu

**Affiliations:** 1 Department of Cardiovascular Surgery, Shanghai Chest Hospital, Shanghai Jiao Tong University, Shanghai, China; 2 Department of Cardiology, Tongji Hospital, Tongji University School of Medicine, Shanghai, China; 3 Quebec Heart and Lung Institute, Laval University, Québec, Canada; 4 The Rugao People’s Hospital, Teaching Hospital of Nantong University, Rugao, China

**Keywords:** Single-cell RNA sequencing, Bicuspid aortic valve, Macrophage, Extracellular matrix remodeling, Valvular calcification

## Abstract

**Background:**

Bicuspid aortic valve (BAV) is the most prevalent congenital valvular heart defect, and around 50% of severe isolated calcific aortic valve disease (CAVD) cases are associated with BAV. Although previous studies have demonstrated the cellular heterogeneity of aortic valves, the cellular composition of specific BAV at the single-cell level remains unclear.

**Methods:**

Four BAV specimens from aortic valve stenosis patients were collected to conduct single-cell RNA sequencing (scRNA-seq). In vitro experiments were performed to further validate some phenotypes.

**Results:**

The heterogeneity of stromal cells and immune cells were revealed based on comprehensive analysis. We identified twelve subclusters of VICs, four subclusters of ECs, six subclusters of lymphocytes, six subclusters of monocytic cells and one cluster of mast cells. Based on the detailed cell atlas, we constructed a cellular interaction network. Several novel cell types were identified, and we provided evidence for established mechanisms on valvular calcification. Furthermore, when exploring the monocytic lineage, a special population, macrophage derived stromal cells (MDSC), was revealed to be originated from MRC1^+^ (CD206) macrophages (Macrophage-to-Mesenchymal transition, MMT). FOXC1 and PI3K-AKT pathway were identified as potential regulators of MMT through scRNA analysis and in vitro experiments.

**Conclusions:**

With an unbiased scRNA-seq approach, we identified a full spectrum of cell populations and a cellular interaction network in stenotic BAVs, which may provide insights for further research on CAVD. Notably, the exploration on mechanism of MMT might provide potential therapeutic targets for bicuspid CAVD.

**Supplementary Information:**

The online version contains supplementary material available at 10.1186/s13062-023-00390-w.

## Background

Calcific aortic valve disease (CAVD) is the most prevalent valve disorder and ranks third among the leading causes of cardiovascular diseases in developed nations [1, 2]. As CAVD progresses, patients typically experience a range of clinical symptoms due to obstruction in the left ventricular outflow tract [3]. Despite the pressing need for effective treatments, there is currently no drug therapy available for severe CAVD. The only options available are surgical valve replacement or transcatheter aortic valve implantation, which highlights the importance of ongoing research into the underlying mechanisms of valvular calcification.

Bicuspid aortic valve (BAV) is the most common congenital valvular heart defect, affecting 1–2% of adults [4]. Over time, most individuals with BAV develop aortic stenosis, and about 50% of severe isolated CAVD cases are identified as BAV [5]. Furthermore, patients with BAV usually develop CAVD earlier and more severely than those with a tricuspid valve (TAV) [6]. Recent studies [7, 8] have demonstrated that mechanisms on the calcification of BAV and TAV are partially similar, but not completely identical. Since the application of scRNA-seq technology in CAVD research [9–12], the heterogeneity of aortic valve cells has received widespread attention. There are notable developmental differences between BAV and TAV that may lead to variations in their cellular composition. Although previous research [9] collected human specimens and provided initial insights into the cell diversity of aortic valves, there is currently no specific study on the cell composition of BAV. Moreover, recent advancements in single-cell sequencing technologies have greatly improved sample preparation and sequencing depth, facilitating the analysis of more cells and genes. Consequently, studying bicuspid CAVD at a single-cell level is imperative for obtaining a comprehensive and in-depth understanding of valve calcification pathogenesis.

Despite numerous efforts to understand how macrophages contribute to aortic valve calcification, prior research has mainly centered around the role of M1-polarized macrophages and their secretion of inflammatory cytokines [13–16]. There may be additional mechanisms by which macrophages promote valvular calcification that have yet to be uncovered, warranting further investigation. The phenomenon of macrophage-to-myofibroblast or mesenchymal transition (MMT) [17] has recently been identified in fibrosis-related diseases affecting multiple organs, such as the lung [18], kidney [19, 20], heart [21], and pancreas [22]. Since there is limited exploration into the differentiation of monocytic lineage [23, 24] in calcific aortic valves, especially the transition of macrophages, it has prompted investigation into the potential involvement of MMT in the progression of valvular calcification.

To gain a more complete understanding of the heterogeneity and investigate the potential developmental transitions of monocytic cells in bicuspid CAVD, we conducted single-cell RNA sequencing (scRNA-seq) on four human stenotic BAV samples with varying levels of calcification. The aim of this study is to provide a cell atlas of stenotic BAV and attempt to reveal novel mechanisms of macrophage involvement in BAV calcification.

## Methods

### Human aortic valve collection

The study recruited 11 patients who underwent aortic valve replacement surgery for bicuspid aortic valves (BAV) (Supplementary Table 1). Single-cell RNA sequencing (scRNA-seq) was performed on specimens collected from 4 of these patients (Supplementary Table 1). To objectively evaluate the extent of valve calcification, Computed Tomography-Aortic Valve Calcium (CT-AVC) scoring was performed using IntelliSpace Portal (Philips). The remaining seven specimens were reserved for histological analysis (Supplementary Table 2).

### Preparation of single-cell suspensions

To isolate cells from the human aortic valve specimens, the tissues were first minced into pieces no larger than 1 mm^3^ and large calcified nodules are removed (Supplementary Video 2). Then tissues were transferred to a solution of 5 ml of digestion medium. This medium consisted of PBS, 0.5 mg/ml collagenase type I (from Sigma-Aldrich), and 0.5 mg/ml collagenase type II (from Sigma-Aldrich). The samples were then incubated at 37 °C for 30 min, with manual shaking occurring every 5 min. Following incubation, the samples were vortexed for 10 s and pipetted up and down for 1 min using a 5 ml pipette. Subsequently, 15 ml of ice-cold PBS containing 0.04% Bovine Serum Albumin (BSA, from Thermo Fisher Scientific) were added, and the samples were filtered through a 70 mm cell strainer (Thermo Fisher Scientific). The undigested calcification fragments were discarded, and the pelleted cells were washed with red blood cell lysis buffer (Miltenyi Biotec) to eliminate any residual red blood cells. After washing with PBS containing 0.04% BSA, the cells were resuspended in the same solution and filtered once more through a 40 μm cell strainer. The dissociated single cells were then stained with Calcein-AM (from Thermo Fisher Scientific) to assess viability, and further enriched with a MACS Dead Cell Removal Kit (Miltenyi Biotec).

### scRNA sequencing

The scRNA-seq libraries were generated according to the protocol of the GEXSCOPETM Single-Cell RNA Library Kit (Singleron Biotechnologies, Nanjing, China). In brief, mixed single-cell suspensions were loaded onto the microfluidic devices, and barcode beads were added after the cells had settled into the wells. The cells were then lysed for 20 min to extract the mRNA, which was captured by the barcode beads. The beads were washed with 6× SSC twice, and the mRNA was reverse transcribed. The cDNA-coated beads were amplified via PCR using the following conditions: 98 °C for 45 s, [98 °C for 20 s, 67 °C for 30 s, and 72 °C for 1 min] x 14 cycles, 72 °C for 1 min. The amplified DNA was purified and assessed for quality using the Agilent BioAnalyzer and a high-sensitivity chip. The purified cDNA was then pooled and subjected to standard Nextera tagmentation and amplification reactions (Nextera XT, Illumina) with a custom primer instead of an i5 index primer, allowing for amplification of only those fragments containing cell barcodes and UMIs. The libraries were sequenced on an Illumina HiSeq X with 150-bp paired-end reads.

### scRNA-seq analysis of BAV

The raw scRNA-seq data was processed using CeleScope software (version 1.4.0, Singleron Biotechnologies). The reads were demultiplexed and mapped to the GRCh38 human reference genome, and the unique molecular identifier (UMI) counts were determined. The low-quality reads were removed and adaptor sequences were filtered using fastp [25] with default parameters to obtain clean data. The cell barcode whitelist was identified through single-cell transcriptome analysis using UMI-tools [26]. The clean UMI-based data was mapped to the human genome (Ensemble version 91) using STAR [27] mapping with customized parameters from the UMI-tools standard pipeline to obtain the UMI counts for each sample. Cells with over 200 expressed genes and a mitochondrial UMI rate below 40% were deemed to have passed the cell quality filtering.

The Seurat package (version 4.0.1, available at https://satijalab.org/seurat/) in conjunction with R software (version 4.0.4) was utilized for the normalization of cells and regression based on the expression table, taking into account the UMI counts and the percentage of mitochondria in each sample to generate scaled data. Quality control visualization was provided in the supplementary material (Supplementary Fig. 2 ). To mitigate the impact of batch effects, we employed SCTransform [28] and integrated the data using the Seurat guideline. An unsupervised cell cluster result was generated using the graph-based clustering method (resolution = 0.8) based on the top 50 principal components obtained from PCA. The marker genes were identified using the FindAllMarkers function with the Wilcox rank sum test algorithm, applying the following criteria: log2FC > 0.25, p-value < 0.05, and min. pct > 0.1. To identify the detailed cell types, the clusters of monocytic cells were selected for further analysis, including re-UMAP analysis, graph-based clustering, and marker gene analysis. Subsequently, to resolve subclusters more effectively, we performed clustering with different resolutions following the above workflow.

### scRNA-seq analysis of previously published dataset

We obtained raw scRNA-seq data on human aortic valve previously published [9]. Using the Cell Ranger toolkit (v6.0.1), we mapped sequencing libraries to the human genome (build GRCh38). We then filtered single cells based on two quality measures: mitochondrial gene expression (< 10%) and gene count (ranging from 200 to 5,000), as determined by the R package Seurat. Basic analysis was performed using the official tutorial, where the top 30 principal components (PCs) were used to perform UMAP reduction and cell clustering. T The resolution of clustering was set to 0.3, and cell types were assigned based on known marker gene expression, taking into account significantly expressed genes in each cell cluster. Further clustering of monocytic cells used same parameters.

### RNA velocity analysis

For RNA velocity [29] analysis, we first generated the bam files using the CeleScope software (version 1.4.0). Then scVelo (https://scvelo.readthedocs.io/) pipeline was used to estimate RNA velocity. A PAGA velocity graph was generated using default parameters to provide a graphical representation of the data topology, with edges weighted according to the connectivity between clusters.

### Pseudotime trajectory analysis

Single-cell trajectory analysis was performed using Monocle2 [30] (version 2.4.7). Dimensionality reduction for pseudotime estimation was performed using the DDRTree method, implemented in the “reduceDimension” function. Prior to Monocle analysis, marker genes were selected from the Seurat clustering results and raw expression counts for each cell were filtered. The root state was manually determined based on RNA velocity results to calculate pseudotime. The pseudotime analysis was followed by Branch Expression Analysis Modeling (BEAM Analysis) to determine gene expression in different branches.

### Cell-cell communication analysis

The R package Cellchat [31] (version 1.6.1) was utilized to infer and analysis cell-cell communications among different cell clusters. We utilized the built-in plotting functions of Cellchat to visualize the communication patterns between various cell types in our dataset, following the tutorials provided on the official website (http://www.cellchat.org/). The database of receptors and ligands used in the analysis was limited to secreted signaling and ECM signals and only included interaction with documented evidence.

### SCENIC analysis

To evaluate the strength of transcription factor regulation, we utilized the single-cell regulatory network inference and clustering [32] workflow (pySCENIC, version 0.10.1) and the 20-thousand motifs database for RcisTarget and GRNboost.

### Enrichment analysis

To identify the potential biological functions of cell clusters, we performed gene set enrichment analysis (GSEA) with marker genes for cell clusters. Marker genes detected in > 25% cells in a cluster with average log2FC > 0.5 were used in the analysis. Enrichment analyses were performed using R package ClusterProfiler (Version 3.14.3). Over-representation tests on Gene Ontology (GO) Biological Processes terms were performed using ClusterProfiler function enrichGO. Genes associated with each term were retrieved from R package org.Hs.eg.db (Version 3.10.0) according to the GO ID of the term.

### Cell culture and MRC1^+^Mac induction

The U937 cell line was originally obtained from American Type Culture Collection (ATCC). Cells were maintained in RPMI 1640 medium containing 10% Fetal Bovine Serum (FBS) (growth medium, GM) at 37 °C in a humidified atmosphere with 5% CO_2_. Osteogenic medium (OM) was used as calcification microenvironment to induce transdifferentiation.

U937-derived MRC1^+^Mac were generated as a macrophage model [33]. Briefly, U937 cells were primed with PMA (Sigma, 10ng/ml) for 48 h to become unpolarized macrophages. To establish the MRC1^+^Mac, the unpolarized macrophages were stimulated with IL-4 (Absin, 20 ng/ml) of for an additional 48 h. Cells were then harvested for flow cytometry analysis (Supplementary Material, Figure S10A) or fixed for immunofluorescent staining (Supplementary Material, Figure S10B) of the indicated markers to examine induction efficiency.

### Flow cytometry

Flow cytometry was used to analyze the effectiveness of macrophage polarization. Briefly, 10^6^ cells were incubated with fluorescent-specific antibodies at 4 °C for 30 min in 100 µl PBS containing 10% fetal bovine serum. APC-conjugated anti-MRC1 antibody (BD) were used to identify MRC1^+^Mac. Labeled cells were washed and analyzed using a BD LSRFortessa flow cytometer and FlowJo software (Tree Star, Inc., USA).

### Histopathology and multiplex fluorescence immunohistochemical staining

Each aortic valve specimen used in histopathology analyses was divided into three parts: fibrotic areas, calcific areas and ILH areas. The degree of calcification in the above areas increased in sequence, as previously described [34]. All parts of the specimens were fixed in 4% paraformaldehyde and decalcified with 10% EDTA. After the tissues were embedded in paraffin, 4 μm sections were prepared using a Leica microtome.

The aortic valve sections were stained according to the instructions of four-color multiplex fluorescence immunohistochemical staining kit (Absin) and blocked with TBST containing 5% goat serum before incubation with antibodies. The antibodies involved in experiments include MRC1 (CST, 24,595 S), α-SMA (abcam, ab240654) and BMP2 (abcam, ab214821). The nuclei were stained with DAPI before sealing, and all sections were scanned by a fluorescent scanning camera (KFBIO, KF-TB-400). Inset images were captured by a confocal microscope. The areas of tissue in sections were calculated using image J according to the scale bars and numbers of positive cells were counted manually. Then the results were quantified as cell densities (cell numbers / tissue areas).

To perform z-stack scans of aortic valve tissue, 20 μm sections were prepared. Images were captured using a confocal microscopy (Zeiss LSM 710). 3D reconstruction was built in Zen2012 software (Zeiss).

### Cell immunofluorescent staining

MRC1^+^Mac cultured in OM or GM were fixed with 4% PFA for 30 min, blocked with 1% BSA for 1 h at room temperature and then reacted with antibodies: α-SMA (abcam, ab240654), MRC1 (CST, 24,595 S) overnight at 4 °C. After washing the cells with PBS, secondary antibodies were applied for detecting above markers. Finally, the cells were counterstained with DAPI (Thermo) and observed using fluorescence microscopy (Zeiss).

### Calcium measurement assay

The intracellular calcium concentration was determined using a Calcium Colorimetric Assay kit (Beyotime, China). The process involved collecting the MRC1 + Mac cells after different times of OM induction, lyzing them with lysate buffer, adding 75 µL Chromogenic Reagent and 75 µL Calcium Assay Buffer to the 50 µL cell lysates, and incubating for 10 min. The absorbance was then measured at 575 nm using a 96-well microplate reader (TECAN, Switzerland). The protein levels were quantified using a bicinchoninic acid assay (Beyotime, China) and the relative calcium levels were calculated by dividing the calcium concentrations by the protein levels.

### Alizarin red staining

After culture in different conditions, cells were washed three times with PBS and fixed with 4% PFA for 30 min, and then stained with 2% Alizarin Red S (Sciencell) for 30 min at room temperature. After washing with deionized water until no more color appearing, the staining pictures were captured. Then the calcium deposits were dissolved in 5% Formic acid and the absorbance value was measured at 450 nm for the quantitative analysis.

### Real-time quantitative PCR

Samples were harvested using Trizol reagent (Invitrogen, Carlsbad, CA) and subjected to RNA isolation. The cDNA was synthesized from the isolated RNA using the RevertAid First Strand cDNA Synthesis Kit (Thermo Fisher Scientific, Waltham, MA). The reverse transcription product was then used as a template for real-time polymerase chain reaction (PCR) performed on a Step One Plus thermal cycler (Applied Biosystems, Foster City, CA) using PowerUp SYBR Green Master Mix (Applied Biosystems). The primer sequences used in the PCR reactions are listed in supplementary Table 3. The final data were analyzed using the 2^-ΔΔc^t method.

### Western blot analysis

The proteins were extracted from the samples using radioimmunoprecipitation assay lysis buffer and the protein concentration was determined using a Bicinchoninic Acid Protein Assay kit. Then, 40 µg of protein from each group were separated by 10 or 12% SDS-PAGE and transferred onto polyvinylidene fluoride (PVDF) membranes. The PVDF membranes were blocked with 5% skim milk for 2 h at room temperature and then incubated overnight at 4 °C with specific antibodies for TIMP1, TIMP2, MMP2, MMP9, Osteopotin, RUNX2, α-SMA, S100A4, FOXC1, PI3K, AKT, p-AKT, and GAPDH. The membranes were then incubated with corresponding horseradish peroxidase-labeled secondary antibodies for 45 min at 37 °C. Finally, the target bands were visualized using an enhanced chemiluminescent kit and analyzed using Image Lab software (Bio-Rad Laboratories).

### siRNA transfection

Prior to transfection, the U937 cells were induced to exhibit the MRC1^+^Mac phenotype. The cells were plated at a density of 4 × 10^5^ cells per well in a 6-well plate. The medium was replaced with fresh serum-free medium one hour prior to transfection. A quantity of 100 pmol of FOXC1 siRNA (siFOXC1 sequence: 5’-CCACTGCAACCTGCAAGCCAT-3’) or negative control siRNA (si-NC) was introduced into the cells using Lipofectamine® 2000 (Invitrogen; Thermo Fisher Scientific). After being incubated at 37 °C for 4 h, the medium was replaced with fresh cell medium containing 10% FBS. The efficiency of silencing was then evaluated through RT-qPCR analysis performed 48 h post-transfection or western blotting performed 72 h post-transfection.

### Statistical analyses

Single-cell RNA sequencing was employed to analyze differential gene expression, and the statistical analysis was performed using the Seurat’s FindMarkers or FindAllMarkers functions in R software. A bimodal likelihood estimator test, which is appropriate for zero-inflated single-cell RNA sequencing data, was utilized. The results are presented as p-values corrected for multiple testing using the Bonferroni correction method.

For single-cell RNA Sequencing statistical analysis of differential gene expression was performed (in R software) with Seurat’s FindMarkers or FindAllMarkers functions by using a bimodal likelihood estimator test suitable for zero-inflated scRNA-seq data. Data is always shown in p-values corrected for multiple testing (Bonferroni).

For Western blots and qPCR data, ANOVA non-parametric Kruskall–Wallis multiple comparison test with posthoc Dunn’s test was used for comparison of multiple groups or a two-sided Student’s t test with Welch’s correction. Statistical testing and plotting were conducted using GraphPad Prism (version 9.4.0). Quantitative data are presented as the mean and SD (mean ± SD). A value of p < 0.05 was considered statistically significant. *p < 0.05; **p < 0.01; ***p < 0.001.

## Results

### Major cellular constitution of human stenotic BAV tissues deciphered by scRNA-seq

**Fig. 1:**
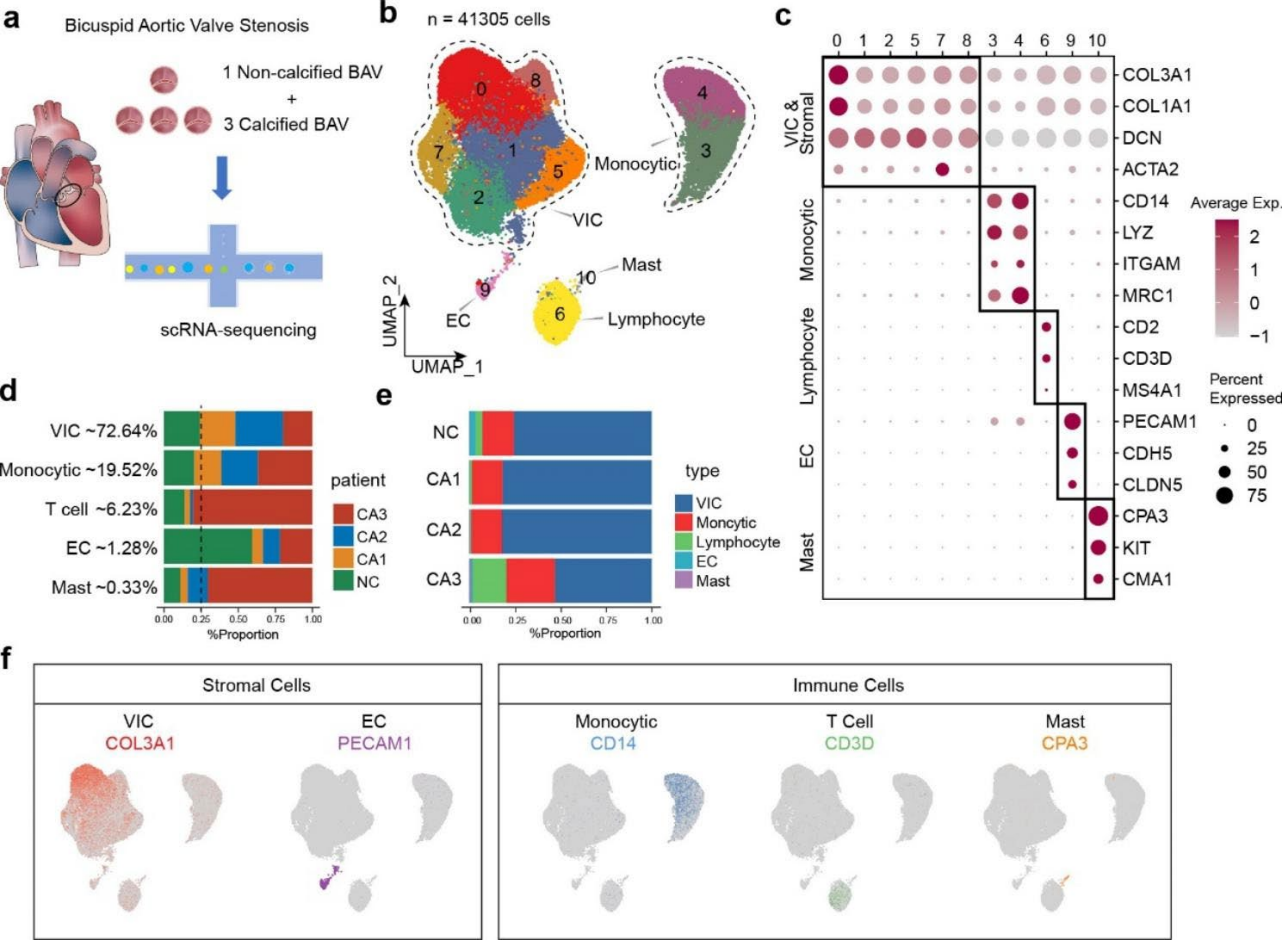
scRNA-seq and major cellular constitution of human stenotic BAV tissues. **(a)** scRNA-seq experiment used 4 specimens (1 non-calcified and 3 calcified specimens) surgically removed from aortic valve stenosis patients with BAV. **(b)** UMAP plot from scRNA-seq data of 4 specimens surgically removed from aortic valve stenosis patients. Data represent n = 41,305 cells. We found 5 major cell types: valvular interstitial cells (VIC), monocytic cells, lymphocytes, endothelial cells (EC), mast cells (Mast). **(c)** Expression patterns of selected markers to annotate major cell types. **(d-e)** The distribution of each subcluster in specimens. **(f)** Important markers of major cell types presented as feature plot (COL3A1 for VIC, PECAM1 for EC, CD14 for monocytic cells, CD3D for lymphocytes and CPA3 for mast cells)

In order to determine the composition of the cell populations in human stenotic BAV tissue, scRNA-seq analysis was conducted on aortic valve tissue samples from one patient with non-calcified valve (NC) and three patients with calcific valves (CA1, CA2, CA3) (Fig. 1a). All patients underwent valve replacement surgery for aortic valve stenosis with/without regurgitation. The clinical information as well as calcium scores of all the specimens is shown in Supplementary Table 1. Specimen NC showed thickened leaflets without calcification, while specimen CA3 got highest score of calcification (Supplementary Table 1 , Supplementary Fig. 1a). After undergoing standard data processing and quality control (Supplementary Fig. 2), 41,305 single cell transcriptomes were obtained (Fig. 1b). The numbers of cells in specimen NC, CA1, CA2, CA3 were 9587, 8810, 11,755 and 11,153, respectively (Supplementary Fig. 1 b-c). In order to acquire unbiased clusters of all cells, we performed sctransform [35] and then integrated data sets across 4 specimens using Seurat [28]. Results showed that batch effects across different specimens were remarkably reduced (Supplementary Fig. 3 a).

After preliminary clustering, 11 clusters were identified based on uniform manifold approximation and projection (UMAP) analyses [36] (Fig. 1b). Top 5 marker genes of each cluster was shown in Supplementary Fig. 3 b. Based on the highly expressed genes of each cluster, we next assigned the 11 clusters into 5 major cell types with typical cell markers to match the biological annotation (Fig. 1c-f, Supplementary Fig. 3 c). In particular, cell markers for cell types were as follows: COL3A1, COL1A1, DCN for VICs (cluster 0, 1, 2, 5, 7, 8); CD14, LYZ, ITGAM for monocytic cells (cluster 3, 4); CD2, CD3D for lymphocytes (cluster 6); PECAM1, CDH5, CLDN5 for endothelial cells (ECs) (cluster 9); CPA3, CMA1, KIT for Mast cells (cluster 10) [23, 24, 37]. The expression differences of the selected marker genes in each cell type were demonstrated through statistical quantification (Fig. 1c).

In our specimens, VICs (72.64%) accounted for the highest proportion among all cell types, followed by monocytic cells (19.52%), lymphocytes (6.23%), ECs (1.28%) and Mast cells (0.33%) (Fig. 1d). We compared the distribution of each cell type within different specimens (Fig. 1d-e). The number of VICs had little variation among 4 specimens. Monocytic cells slightly increased, while lymphocytes and mast cells significantly rose in ca03, which was the severely calcific specimen. In consistent with previous research, the proportion of monocytic cells was much higher than other immune cell types.

In particular, we have identified the presence of mast cells in BAV, which was not previously reported in single-cell studies of CAVD [9]. Histological studies in recent years have also identified CD117^+^ mast cells [38], but their involvement in CAVD is poorly understood. Our sequencing results show that the number of mast cells is very low, accounting for only 0.33%, and their role in valve calcification requires further investigation.

### VIC and stromal cells exhibited significant heterogeneity

**Fig. 2:**
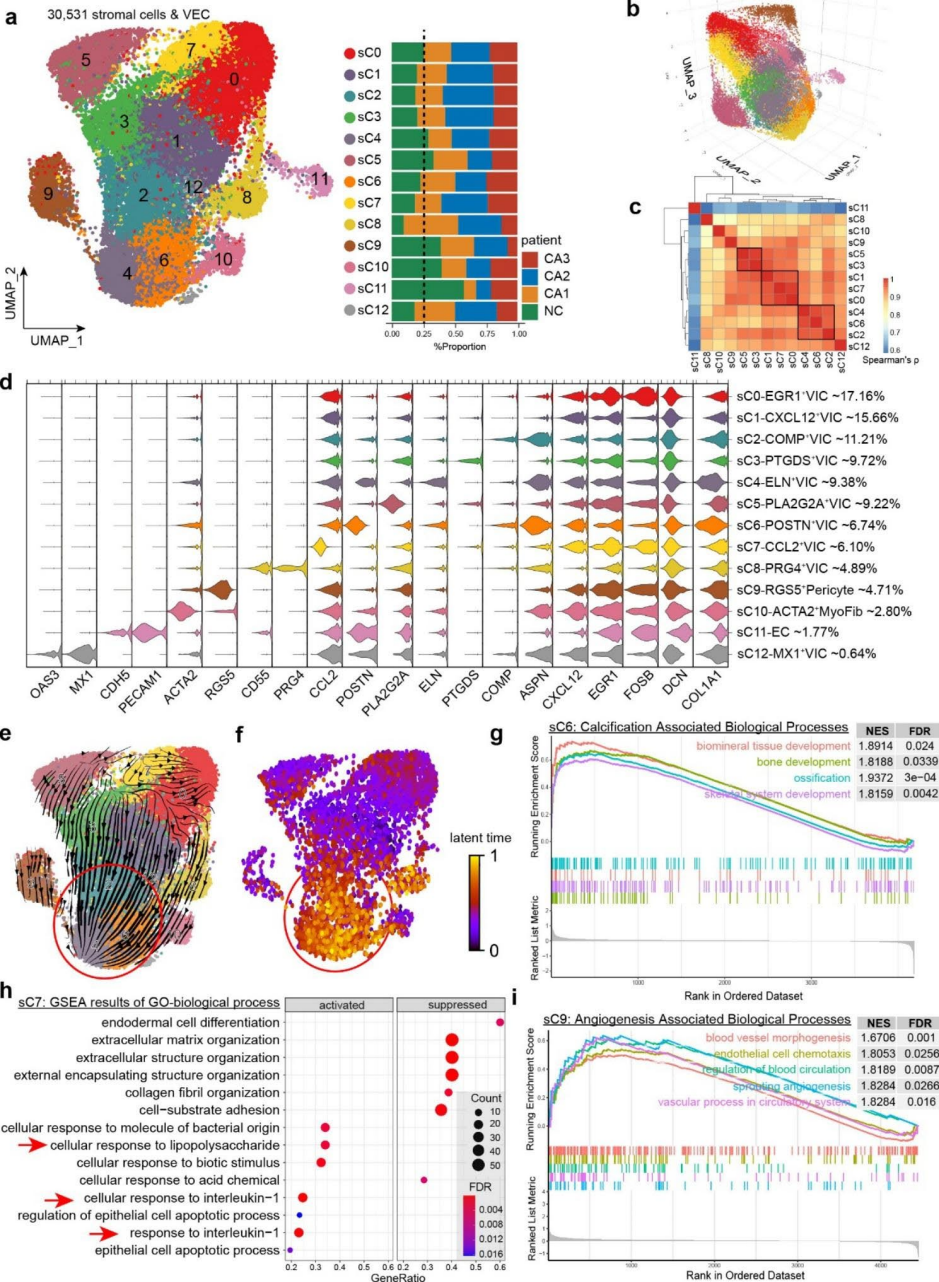
Subcluster of stromal cells in BAV. **(a)** UMAP projection showing 13 subclusters of stromal cells (VICs and EC), and cell distribution in different specimen. Data represent n = 30,531 stromal cells. **(b)** Visualization of 3D projection of UMA plot. **(c)** Spearman correlation analysis based on top 1000 highly variable genes. **(d)** Stacked violin plot showing selected marker genes of each monocytic subcluster. **(e)** RNA velocity plot of stromal cells and **(f)** cellular latent time calculated via dynamic modeling. **(g)** GSEA plots indicated calcification associated processes were activated in POSTN^+^VIC (sC6). **(h)** Dot plot of GSEA results indicated response to interleukin-1 were activated in CCL2^+^VIC (Sc7). **(i)** GSEA plots indicated angiogenesis associated processes were activated in RGS5^+^VIC (sC9)

Based on UMAP analyses, we further identified 13 cell populations among 30,531 stromal cells (VIC & EC) by UMAP analysis (Fig. 2a). 3-dimentional (3D) UMAP reduction with spatial structures was demonstrated to help us further explain the heterogeneity among these cells (Fig. 2b). Stromal cells, especially VIC, had great complexity. Top 5 markers of each cluster were shown in Supplementary Fig. 4 . Correlation plot of subclusters (sC) based on top 1,000 highly variable genes demonstrated similarities among subclusters (Fig. 2c).

We selected specific cell markers for each subcluster from their highly expressed genes (Fig. 2d). We first discovered EGR1^+^VIC (sC0), the largest subpopulation in VICs. Upon examining its gene expression, we found high expression levels of EGR1, FOB, and HSP family proteins, which are characteristic proteins of early responses, indicating that these cells are activated VICs. In contrast, the adjacent CXCL12^+^VIC (sC1) exhibited a static fibroblast-like expression profile without specific upregulated molecules, and we referred to them as static VICs.

Populations sC2, sC4, sC6 and sC8 had significant expressions of collagens and ASPN, an osteo/chondroblastic marker [39], were defined as COMP^+^VIC, ELN^+^VIC, POSTN^+^VIC and PRG4 + VIC respectively. Since POSTN (periostin) and COMP (chondrogenic oligomeric protein) are important ingredients in bone and cartilage, these populations might be the osteoblastic or chondroblastic VICs in calcific valves. RNA velocity analysis using a dynamical model [29] served as additional evidence supporting the assumption: COMP^+^VIC, ELN^+^VIC and POSTN^+^VIC distributed at the end of the arrows (Fig. 2e, Supplementary Fig. 5 ), and their latent time was the highest (Fig. 2f), which indicated that they were probably transdifferentiated from other VICs. Gene set enrichment analysis (GSEA) of POSTN^+^VIC revealed that calcification-associated biological processes, including ossification and bone development, were significantly activated (Fig. 2g). These subclusters (sC2, sC4, sC6 and sC8) were defined as Osteoblastic VIC.

The sC10, defined as ACTA2 + MyoFib, was found to exhibit elevated expression levels of collagens and ASPN. Consistent with the current understanding of CAVD, which suggests that myofibroblast/osteoblast differentiation of VICs is a key mechanism underlying valve calcification, RNA velocity analysis indicated that ACTA2 + MyoFib was also one of the major destinations of VICs transdifferentiation (Fig. 2e-f).

There are several newly discovered populations as well. GSEA showed that sC0, sC1, sC3, sC5, sC7, as well as sC12 were activated with different kind of immune response (Fig. 2h, Supplementary Fig. 6 ). For example, cellular responses to interleukin are activated in CCL2^+^VIC (sC7), and PTGDS^+^VIC (sC3) highly expressed prostaglandin D2 synthase. Interestingly, PLA2G2A^+^VIC (sC5) was found to be involved in both lipid metabolism and immune response, as evidenced by its high expression of phospholipase and upregulated immune response pathways (Supplementary Fig. 6 ). These populations are inflammatory VICs (sC0, sC1, sC3, sC7, sC12) and lipid metabolic VIC (sC5).

Our findings also revealed a previously unidentified population of pericytes (sC9) in BAV, identified by the expression of the typical pericyte marker RGS5. These pericytes may potentially play a role in angiogenesis within the aortic valves. Enrichment results also indicated that angiogenesis-associated processes are significantly activated in RGS5^+^Pericyte (Fig. 2i). Many researchers have noticed the phenomenon of neovasculature and intraleaflet hemorrhage (ILH) [40, 41] in severely calcified aortic valves. The specific mechanism of neovascularization in the valve is still unclear, but recent studies have shown that VICs can promote endothelial proliferation and vascular formation in vitro, similar to the function of pericytes [42]. However, it was unclear whether pericytes exist and what their role was in the human valves. In our study, we identified pericytes as a distinct subpopulation of in BAV.

### Functional diversity of endothelial cells in BAV

**Fig. 3:**
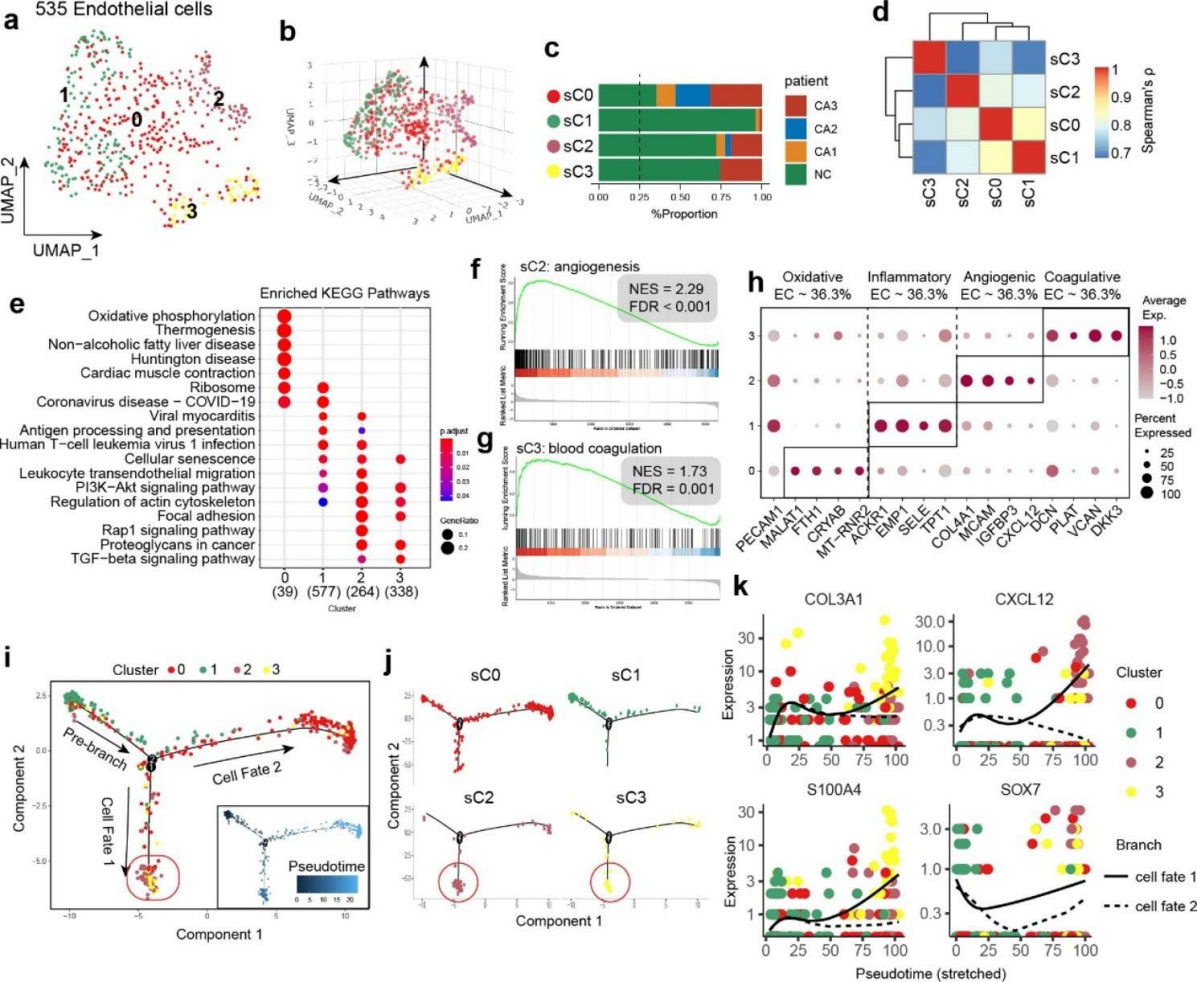
Functional diversity of endothelial cells. **(a)** UMAP plot showing 4 subclusters of EC. Data represent n = 535 ECs. **(b)** Visualization of 3D projection of UMAP plot. **(c)** The distribution of each subcluster in specimens. **(d)** Spearman correlation analysis based on top 1000 highly variable genes. **(e)** Enriched KEGG pathways in different subclusters. **(f)** GSEA plots showing biological process of angiogenesis was activated in sC2. **(g)** GSEA plots showing biological process of blood coagulation was activated in sC3. **(h)** Dotplot showing selected marker genes of each EC subcluster. **(i-j)** Trajectory and pseudotime of ECs on DDRTree projection (inferred using Monocle2). **(k)** EndMT related genes were upregulated in cell fate 1

The integrity of the aortic valve endothelium is an important factor in maintaining valve homeostasis [43]. Endothelial dysfunction is considered one of the initiating factors of CAVD [44], so it is important to clarify the heterogeneity of endothelial cells to deepen the understanding of the relevant mechanisms. We further divided endothelial cells into 4 subpopulations based on UMAP projection (Fig. 3a-b). We found that the number of ECs was highest in the NC group, while it was significantly reduced in the CA group, which may be due to technical bias during sample processing. However, within the calcification group, we found that the more severe the calcification, the greater the number of endothelial cells (Fig. 3c), which is consistent with our existing knowledge of neovascularization in the valve.

Top 5 marker genes of each subcluster were presented in Supplementary Fig. 7. The correlation heatmap (Fig. 3d) shows that the 4 subclusters of ECs have high overall correlation (> 0.7). Pathway analysis (Fig. 3e) showed that the oxidative phosphorylation pathway was highly activated in sC0. Combined with its high expression of many mitochondrial-related genes (Supplementary Fig. 7 ), it was regulated by oxidative stress. Therefore, we defined sC0 as the oxidative EC. The sC1 exhibited high expression of genes such as ACKR1, SELE, and EMP1, many of which are crucial adhesion molecules on the surface of ECs (Fig. 3h, Supplementary Fig. 7 ). These molecules play important roles in the adhesion, chemotaxis, and antigen presentation processes between endothelial and immune cells. This subcluster can be classified as inflammatory EC.

The involvement of EC in oxidative stress regulation and immune activity has been extensively studied. While our analysis of sC2 and sC3 provided new insights into the role of VEC in valve calcification.

On the UMAP projection, sC2 and sC3 are located far from the main body of ECs, indicating that these two subgroups may undergo phenotypic transformation (Fig. 3a-b). The KEGG pathway analysis showed that the signaling pathways regulating these two subclusters had similarities, as we found that the PI3K-AKT and TGF beta signaling pathways were both activated (Fig. 3e). These two pathways are key signals regulating cell differentiation and EndMT. GO enrichment analysis revealed that these two subclusters of ECs have independent functions, with sC2 participating in angiogenesis and sC3 mainly involved in blood coagulation (Fig. 3f-g, Supplementary Fig. 8 ). Therefore, we identified them as angiogenic EC and coagulative EC.

The inflammatory EC (sC1) were almost exclusively found in NC samples and rarely observed in calcified group, indicating that this subcluster can serve as the starting point for EC phenotype transformation. Based on this, we utilized Monocle2 for trajectory analysis (Fig. 3i-j) and found that sC2 and sC3 formed the same cell fate (cell fate 1). Although they have functional differences, they may have undergone similar transformation processes. Further analysis revealed that EndMT-related genes were significantly upregulated during the cell fate 1 transition as determined by pseudotime analysis (Fig. 3k). For example, CXCL12 is a factor derived from stromal cells, while S100A4 (Fibroblast specific protein 1) is a key transcription factor involved in the regulation of the EndMT process.

### Lymphocytes significantly increased in severe calcification

**Fig. 4:**
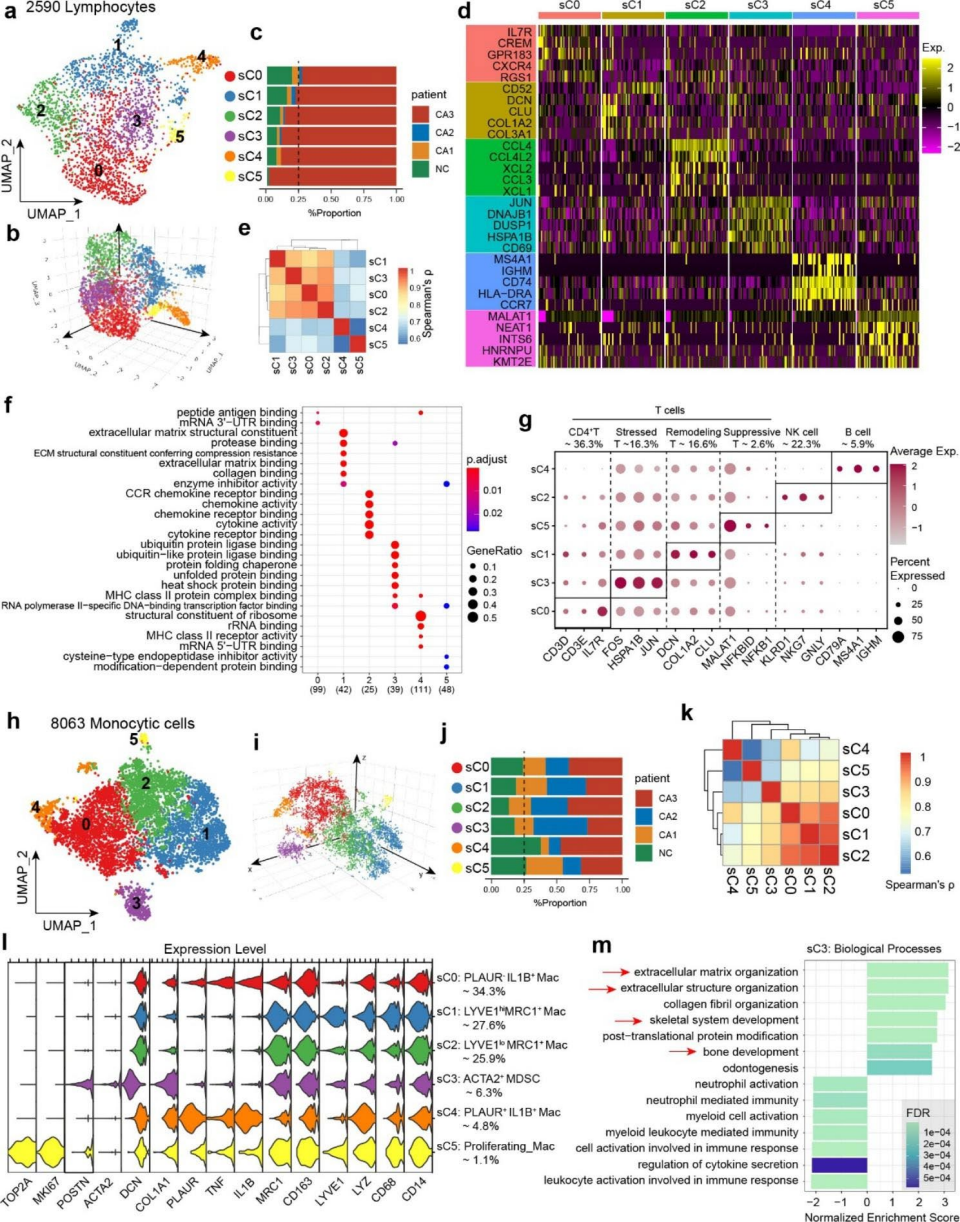
Re-clustering of immune cells in BAV. **(a)** UMAP plot showing 6 subclusters of lymphocytes. Data represent n = 2590 lymphocytes. **(b)** Visualization of 3D projection of UMA plot. **(c)** The distribution of lymphatic subclusters in specimens. **(d)** Top 5 marker genes of lymphatic subclusters generated using Seurat. **(e)** Spearman correlation analysis of lymphatic transcriptomes based on top 1000 highly variable genes. **(f)** GO enrichment analysis of lymphatic subclusters. **(g)** Selected markers for each subcluster represented as dot plot. **(h)** UMAP plot showing 6 subclusters of monocytic cells. Data represent n = 8063 monocytic cells. **(i)** Visualization of 3D projection of UMA plot. **(j)** The distribution of each subcluster in specimens. **(k)** Spearman correlation analysis based on top 1000 highly variable genes. **(l)** Stacked violin plot showing selected marker genes of each monocytic subcluster. **(m)** GSEA plots showing ECM organization and calcification related processes were activated in ACTA2^+^MDSC (sC3)

Immune cells play an important role in the occurrence and development of valve calcification. We first explored the types and functions of lymphocytes in BAV. Based on UMAP projection, we re-analyzed 2590 lymphocytes and obtained 6 subclusters (Fig. 4a-b). Lymphocytes were almost exclusively detected in the severe calcification (CA3) specimen, with low numbers in the NC and mild calcification specimens (Fig. 4c). This suggests that lymphocytes may participate in the remodeling of the immune microenvironment in the late stage of valve calcification, rather than in the early stage.

Top 5 marker genes of each subcluster were presented as a heatmap (Fig. 4d). The correlation heatmap shows that the expression profiles of sC0, sC3, sC1, and sC2 subclusters have some similarities, while sC4 and sC5 differ significantly from other subclusters (Fig. 4e). Both sC0 and sC3 exhibit high expression of IL7R (Fig. 4g), which is a marker of CD4^+^ T cells. Enrichment analysis also indicates that these two cluster of cells are involved in B cell activation (Supplementary Fig. 9) and MHC-II molecule binding (Fig. 4f), indicating their function as helper T cells. The sC3 subcluster shows high expression of FOS, JUN, and HSP family molecules (Fig. 4g), which are markers of stress response, and we refer to these cells as stressed T cells. sC1 was a special T cell population, expressing both T cell marker and stromal markers, including DCN, COL1A2 and CLU. This subcluster was also identified in non-diseased valves [45], as remodeling T cell.

The sC2 subcluster also exhibits strong immune activity, with highly activated chemokine-related signaling pathways (Fig. 4f, Supplementary Fig. 9 ). It showed high expression levels of natural killer (NK) cell markers, including KLRD1, NKG7, and GNLY, indicating that this subcluster is NK cells. The sC4 exhibited high expression of CD79A and MS4A1, which are typical B-cell markers, and was capable of synthesizing immunoglobulin M (IGHM), suggesting it may be partially activated plasma cells (Fig. 4G). We currently identified this subcluster as B cells.

Interestingly, we also found a small population of T cells that were almost exclusively present in specimen with severe calcification. These cells were rare, accounting for only 2.6% of all lymphocytes (Fig. 4g). Unlike other T cell subsets, these cells exhibited immunosuppressive activity and expressed high levels of inhibitory molecules such as NFKB1 and NFKBID. They were likely regulatory T cells, and we here referred to them as suppressive T cells. The emergence of suppressive T cells may be the result of the body’s attempt to achieve homeostatic regulation under high levels of inflammation in severely calcified BAV.

### Subclusters of monocytic cells and macrophage derived stromal cells in BAV

Next, we re-clustered the 8063 monocytic cells into 6 subclusters (Fig. 4h-i). The distribution of all the subclusters in each specimen was shown in Fig. 4j. The spearman correlation of top 1,000 most variable genes indicated sC0, sC1 and sC2 were closely related, while other subclusters seemed to be relatively independent (Fig. 4k). Top 10 marker genes generated by Seurat *FindMarkers* function were demonstrated as a heatmap (Supplementary Fig. 10). We assigned selected marker genes of each subclusters to biologically annotate these subclusters based on their specific highly expressed genes (Fig. 4l, Supplementary Fig. 11).

Firstly, we confirmed all the monocytic subclusters expressing typical monocyte and macrophage markers CD14 and CD68 (Fig. 4l). Among all the subclusters, sC5 was recognized as proliferating macrophages for high expression of cell cycle genes TOP2A and MKI67. sC0 and sC4 were IL1B^+^ Mac (M1-like macrophages), expressing inflammatory cytokines TNF and IL1B. PLAUR is a gene encoding a monocyte activation antigen [46] (Urokinase Plasminogen Activator Surface Receptor, uPAR). sC4 was defined as PLAUR^+^ IL1B^+^ Mac, showing the strongest pro-inflammatory properties among all subclusters. While sC0, rarely expressing PLAUR, was defined as PLAUR^-^ IL1B^+^ Mac (Fig. 4l). IL1B^+^ Macs, especially PLAUR^+^ IL1B^+^ Mac, increased dramatically in severe calcification group compared to mild ones (Fig. 4j). sC1 and sC2 were MRC1^+^ Mac (M2-like macrophages), highly expressing CD163 and MRC1 (alias CD206). The expression profiles of sC1 and sC2 were very similar, with limited differences in expression levels of certain genes. We defined them as LYVE1^hi^MRC1^+^Mac and LYVE1^lo^MRC1^+^Mac respectively (Fig. 4l, Supplementary Fig. 11). LYVE1^lo^MRC1^+^Mac slightly increased in severely calcified valve, while the percentage of LYVE1^hi^MRC1^+^Mac remained stable across 4 specimens (Fig. 4j). In addition, although no osteoclast subpopulation was identified, we observed elevated expressions of osteoclastic markers (CTSK, ACP5) in LYVE1^hi^MRC1^+^Mac (Supplementary Fig. 12).

To our surprise, sC3 was a special monocytic subcluster, expressing both monocytic markers (CD14, CD68 etc.) and mesenchymal markers (COL1A1, ACTA2 etc.). It was defined as ACTA2^+^MDSC (Macrophage-Derived Stromal Cells) (Fig. 4l). Interestingly, GESA results showed biological processes of ECM organization and skeletal system development were significantly activated (Fig. 4m). Therefore, we assumed ACTA2^+^MDSC probably play an important role in the calcification of BAVs. Other GSEA results were organized in Supplementary Fig. 13.

### CD68 and α-SMA co-expressing cells were present in calcific BAVs

**Fig. 5:**
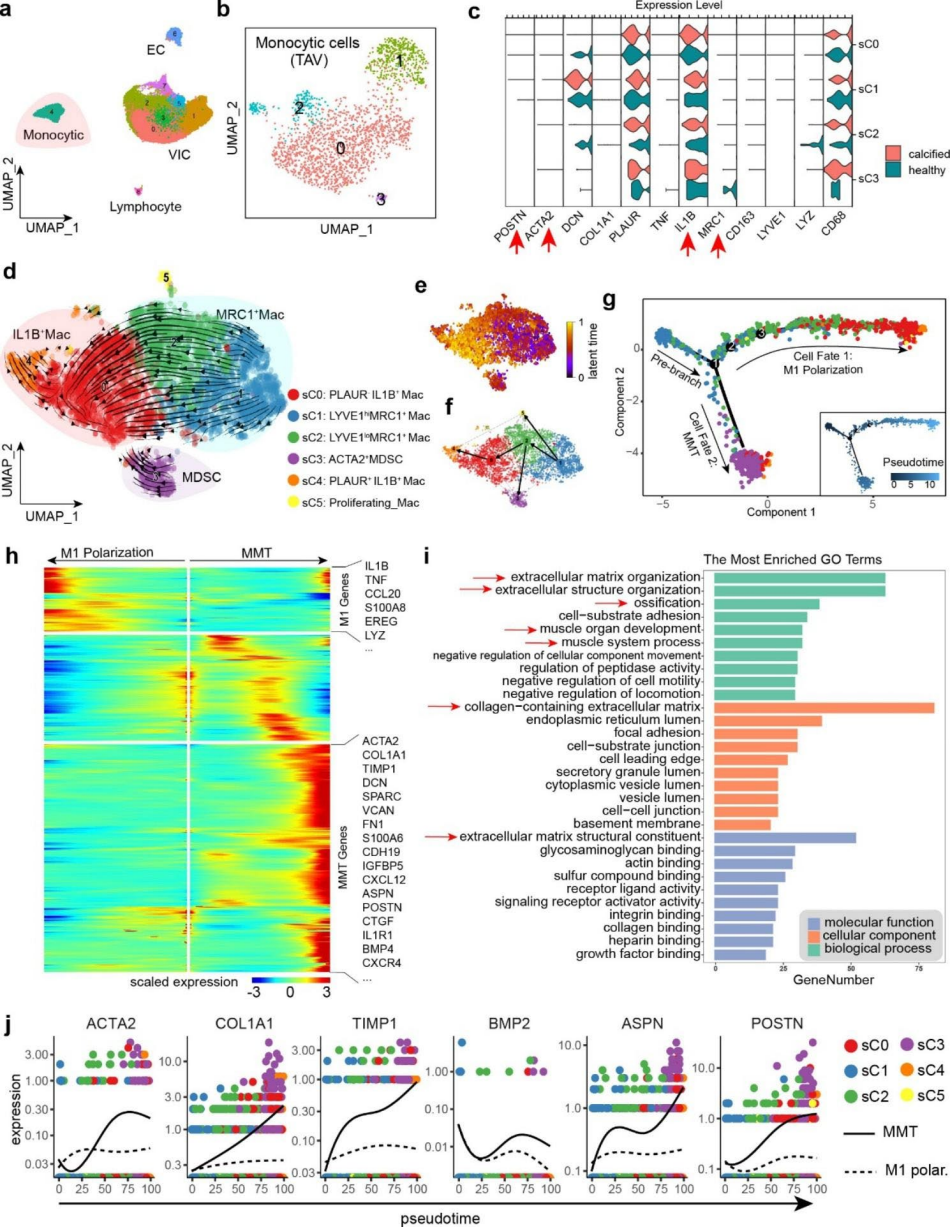
Trajectory analysis identified MMT in bicuspid CAVD. **(a)** a re-analysis of previously published CAVD dataset. **(b)** Subclusters of monocytic cells in previous dataset. **(c)** Examine selected genes in different subclusters of monocytic cells. Data indicated that MDSC subpopulation was not identified in previous CAVD dataset. **(d)** Velocity graph of monocytic cells based on UMAP projection. The arrows on the graph indicates the future states of the cells inferred by RNA velocity. **(e)** Velocity pseudotime plot. **(f)** PAGA graph based on RNA velocity. Lines with arrows indicate high probability of transition. **(g)** Pseudotime trajectory computed by Monocle2. Trajectory showing 3 major branches (1 pre-branch and 2 cell fates). **(h)** Branched heatmap showing gene expressions change as pseudotime goes. DEGs was clustered into 3 groups. **(i)** The group of MMT genes was used to conduct gene ontology (GO) enrichment. ECM organization and ossification processes were enriched. **(j)** The expression dynamics of representative genes in branched cell fates

To identify whether ACTA^+^MDSC really exists in BAV, we conducted tissue immunofluorescence. Results showed that the number of CD68 and α-SMA co-expressing cells was elevated in the calcific and ILH areas (Supplementary Fig. 14), suggesting that MDSC were present in calcific BAVs. To further determine the presence of ACTA^+^MDSCs in TAV, we conducted a re-analysis of a previously published scRNA-seq dataset [9] consisting of two healthy and four calcified aortic valves (Fig. 5a, Supplementary Fig. 15). After we identified major cell types, we re-clustered monocytic cells in the previous dataset (Fig. 5b). The transcriptional expression levels of key genes were visualized in Fig. 5c. Notably, all monocytic cell subclusters expressed IL1B, indicating a strong pro-inflammatory capacity of macrophages, while none of the subclusters were found to express mesenchymal markers. Thus, no MDSC population was identified in this dataset.

### RNA velocity and monocytic lineage trajectory analyses revealed macrophage-to-mesenchymal transition

In this part, we performed RNA velocity on the UMAP projection firstly. The percentages of spliced RNA in each subcluster were shown in Supplementary Fig. 16a-b. In the velocity graph (Fig. 5d-e), LYVE1^hi^MRC1^+^Mac was placed at the start point of monocytic lineage and had a potential of transition to pro-inflammatory state. Based on the directed velocity graph, the velocity pseudotime was computed, which implicitly infers the root cells (Fig. 5d). Plots of velocity length and velocity confidence calculated by scVelo was demonstrated in Supplementary Fig. 16c-d. PAGA graph abstraction [47], a top-performing method for trajectory inference, was conducted to provide a map of the data topology with weighted edges corresponding to the connectivity between two subclusters (Fig. 5f). Results indicated that MRC1^+^Macs possessed the tendency to transdifferentiate to IL1B^+^ Macs and MDSCs.

To further confirm the underlying transition, we then used Monocle2 to rearrange the cells and build another trajectory based on the DDRTree projection (Fig. 5g). It was determined that the cells matched 3 potential differential branch point, but only one point formed a complete branch and separated all cells into 3 distinct states: a pre-branch and two cell fates (Fig. 5g). It was obvious that MRC1^+^Macs, especially LYVE1^hi^MRC1^+^Mac, spread over the pre-branch. The facets of the plot showed that two subclusters of IL1B^+^ Macs gathered at cell fate 1 while MDSC distributed at cell fate 2 (Supplementary Fig. 16e). Consistent with RNA velocity, the results offered profound evidence that MRC1^+^Macs in calcific aortic valves might undergo a two-way transition: classical M1 polarization and MMT.

Interestingly, when we tried to perform a trajectory analysis on the non-calcified specimen (NC) independently, MDSCs did not form an explicit branch in the trajectory (Supplementary Fig. 16f). To study the reasons for this difference, we calculated the differential expression genes (DEGs) of MDSCs (CA vs. NC) and then performed gene ontology analyses (Supplementary Fig. 16g). Mitochondrial respiratory chain was active in calcific valves while biological processes related to immune function were downregulated, and myoblast proliferation genes were upregulated. We supposed that MDSCs in calcific valves evolved into a phenotype with stronger mesenchymal properties through MMT and participated in calcification formation.

Differential gene expression patterns in branched cell fates were analyzed using a clustering heatmap (Fig. 5h). As expected, pro-inflammatory genes gradually increased in M1 polarization (cell fate 1) over pseudotime. The group of genes rising in MMT (cell fate 2) was marked as MMT genes (Fig. 5h). To precisely annotate the biological functions of MMT genes, we applied enrichment analyses of gene ontology (Fig. 5i). MMT genes was highly enriched to ECM organization, ossification, and muscle system process etc. ACTA2 (encoding α-smooth muscle actin, α-SMA), a myofibroblast and EMT (endothelial-to -mesenchymal transition) marker, upregulated when MMT happened. ECM composition COL1A1, ASPN, POSTN significantly increased. Another important gene is TIMP1, a potent inhibitor of Matrix metalloproteinases (MMPs), whose rise will exacerbate ECM accumulation.

Current studies suggest that osteoblast-like cells (OBLC) in calcified valves mainly come from the transdifferentiation of VICs, while recently, a study speculated that M2 macrophages (MRC1^+^Mac) might be one of the sources of OBLC for observing the co-expression of MRC1 and BMP2 in human calcific aortic valves [48]. Such phenotype of macrophages is similar to MDSCs we reported here. Moreover, scRNA-seq profile showed osteogenic markers BMP2, ASPN, POSTN were upregulated as MMT developed, indicating osteogenic potential of MDSCs (Fig. 5j).

### Cellular interaction network within BAV constructed via cellchat

**Fig. 6:**
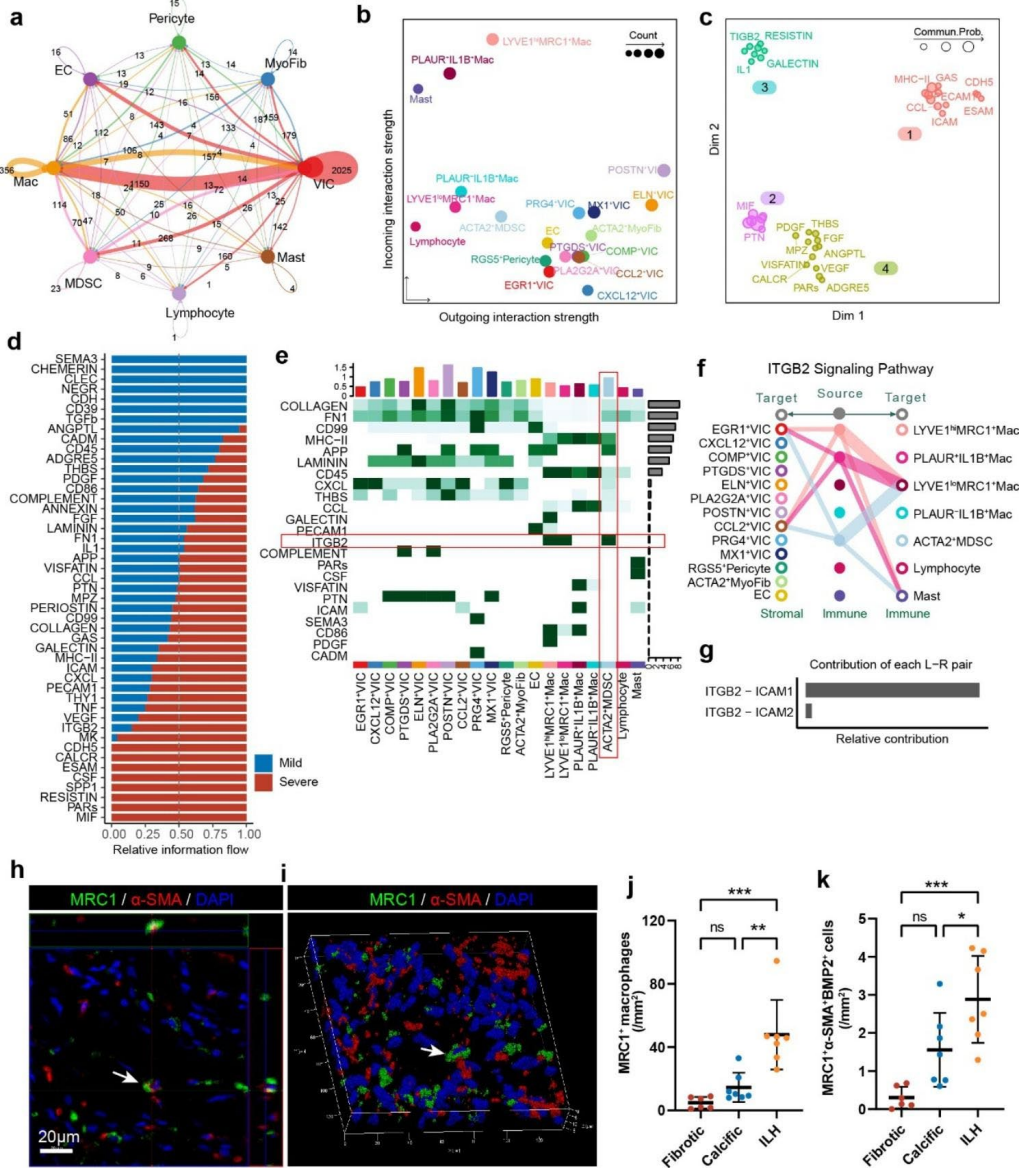
Cellular interaction network conducted using Cellchat. **(a)** Cell-cell communication among major cell types inferred using Cellchat. **(b)** Dotplot showing incoming and outgoing interaction strength of all cell types. **(c)** Principal component analysis showing functional clusters of all signaling pathways. **(d)** Relative information flow (Severe vs. Mild calcification) of all detected cell-cell communications. (**e**) A heatmap of outgoing signal strength from all cell types. ACTA2 + MDSC released strong ITGB2 signal. (**f**) Signaling patterns of ITGB2. ITGB2 signaling from ACTA2 + MDSC can exert strong influence on EGR1^+^VIC (activated VIC) and CCL2^+^VIC (Inflammatory VIC). **(g)** The relative contributions of different L-R (Ligand-Receptor) pairs of ITGB2 signaling pathway. **(h)** The orthographic view of z-stack scans of a calcific aortic valve section showing MRC1 and α-SMA co-expressing cells. (Scale bar: 20 μm) **(i)** The 3D view of a representative section. **(j-k)** Quantitative results of MRC1^+^cells **(j)** and α-SMA^+^MRC1^+^BMP2^+^cells **(k).** Numbers of certain cells were manually counted, areas of sections with tissue were measured using Image J

To better understand the interactions between different cell types in BAV, we performed cell communication analysis using the Cellchat software. The results showed that the cellular interaction network in BAV is highly complex, including interactions between multiple cell types (Fig. 6a). In this network, the autocrine secretion of VICs and the interaction between VICs and macrophages are the most significant, which means VICs and macrophages consisted the core of this network. The network of different cell types involved in different signal interactions are presented in the form of bubble diagrams (Supplementary Fig. 17).

To explore the strength of signaling interactions among different cell subpopulations, we found that osteogenic VICs (POSTN^+^VIC and ELN^+^VIC) had the highest signal strength, while macrophages had the highest signal reception strength, further indicating that VICs and macrophages are the core of the interaction network (Fig. 6b). We also performed clustering analysis of the interaction structures of various pathways in the BAV signaling network, which allows us to infer their potential effects and facilitates further investigation of specific signals in the future (Fig. 6c).

After analyzing each sample individually, we compared the interaction signals between samples with severe and mild calcification. We examined the changes in signal strength in severe calcification to infer their correlation with valve calcification. We found that ICAM, CXCL, PECAM1, THY1, TNF, VEGF, ITGB2, and MK signals were significantly elevated in severe calcification, while CDH5, CALCR, ESAM, CSF, SPP1, RESISTIN, PARs, and MIF signals were almost exclusively detected in severe calcification (Fig. 6d). These data suggest that the aforementioned signals may be associated with the exacerbation of BAV calcification.

### MDSC phenotype was involved in BAV calcification

Our analysis revealed that MDSC exhibited a robust activation of the ITGB2 (integrin β2) signaling pathway (Fig. 6e), which is capable of exerting influence on inflammatory VICs (CCL2^+^VIC), activated VIC (EGR1^+^VIC) and immune cells (Fig. 6f). This interaction primarily based on ITGB2-ICAM1 L-R (Ligand-Receptor) pair (Fig. 6g). Given that dysregulation of integrin signaling is associated with ECM remodeling of VICs [11], it is plausible that the MDSC population may promote the progression of BAV calcification.

To further evaluate the MDSC phenotype, we employed multiplex immunofluorescence to detect the co-expression of α-SMA, MRC1, and BMP2 (Supplementary Fig. 18). Cells exhibiting co-expression of α-SMA, MRC1, and BMP2 were identified as MDSCs, possessing the ability of myofibroblast/osteoblast transdifferentiation. The co-expression of MRC1 and α-SMA was clearly demonstrated in both the orthographic view and 3D reconstruction of z-stack scans. (Fig. 6h-i). Our results revealed a slight increase in both MRC1^+^Mac and α-SMA^+^MRC1^+^BMP2^+^MDSCs in calcified areas, and a significant increase in these cell populations in areas with ILH (Fig. 6j-k).

### Macrophages can adopt a MDSC phenotype in vitro

U937 cells are widely used in CAVD [49, 50] and various macrophage-related research [51, 52]. To investigate whether MRC1^+^ macrophages can acquire a MDSC phenotype in vitro, we firstly induced U-937 cells into MRC1^+^ macrophages (Supplementary Fig. 19a-b) and then stimulated the cells with osteogenic medium (OM) to create a calcification microenvironment.

**Fig. 7:**
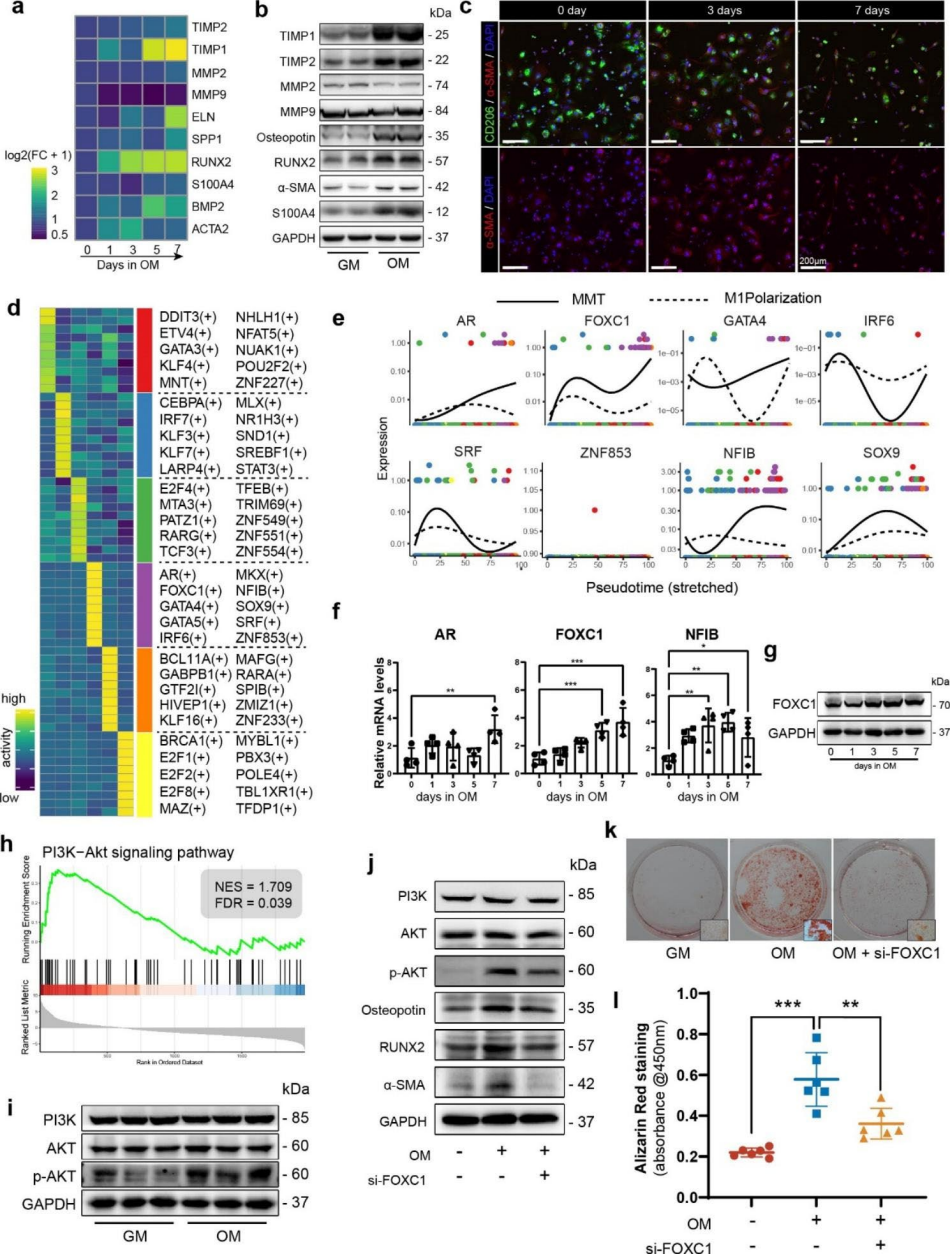
In vitro studies and SCENIC analysis identified crucial regulators of MMT. **(a)** mRNA levels of selected markers (fibro/osteoblastic markers and ECM related proteins). Data was normalized as log2(FC + 1). FC: fold change to control. **(b)** Representative western blot images of fibro/osteoblastic markers and ECM related proteins. MRC1^+^Macs were incubated in OM for 7 days. (n = 5,5 for GM, OM) **(c)** Representative images of immunofluorescence after MRC1^+^Mac incubated in OM for 3 or 7 days. Anti-MRC1: green, anti-α-SMA: red, DAPI: blue. Scale bar: 200 μm. (**d**) Heatmap of the mean value of area under the recovery curve (Aucell) value of expression regulation by the transcription factors, as estimated using SCENIC. The top 10 transcription factors genes (TFs) of each subcluster were shown. (**e**) The expression dynamics of top 10 TFs in MDSC. GATA5, MKX, ZNF853 were hardly detected in transcriptome. (**f**) RT-qPCR examined AR, FOXC1 and NFIB mRNA levels changes after OM incubation of MRC1^+^Mac. (n = 4 for each group) **(g)** The protein levels of FOXC1 in MRC1^+^Mac after OM incubation. **(h)** GSEA plot showing PI3K-AKT signaling pathway is significantly activated in MDSC. **(i)** The phosphorylation levels of AKT upregulated in vitro after OM induction. **(j)** The proteins levels of myofibro/osteoblastic markers and PI3K-AKT pathway after treatments of OM an si-FOXC1 transfection. **(k)** Representative images of alizarin red staining of each group. **(l)** The quantitative results of alizarin red staining (n = 6)

After 7 days of osteogenic medium (OM) induction, we evaluated mRNA levels (Fig. 7a) and protein levels (Fig. 7b, Supplementary Fig. 19c-j) of crucial molecules and presented quantitative analysis results. The mesenchymal marker S100A4 was found to be elevated (Supplementary Fig. 19c). Myofibroblastic marker (α-SMA) and osteogenic markers (RUNX2 and Osteopontin) were significantly upregulated (Supplementary Fig. 19d-f). MMPs and TIMPs are important regulators of ECM organization, and the imbalance of their system has been reported to be positively associated with CAVD [53–55]. We found that MMP9 expression level mildly decreased (Supplementary Fig. 19j), while MMP2 expression level showed no differences between groups (Supplementary Fig. 19i). However, both MMPs potent inhibitors TIMP1 and TIMP2 (Supplementary Fig. 19g-h) were upregulated obviously. Collectively, these results suggest that not only did MDSCs possess the potential of myofibro/osteoblastic transition, but they also aggravated ECM accumulation.

Furthermore, we discovered that there was a correlation between the length of incubation time and the morphology of MRC1^+^Mac. Specifically, we observed a transformation from a macrophage with typical pseudopodia to a spindle-shaped stromal morphology (Fig. 7c, Supplementary Fig. 19k). To provide concrete evidence of calcium deposition, we conducted calcium measurement assays to quantitatively assess the levels of cellular calcium. Our results indicated that calcium content gradually increased as the induction days in OM increased (Supplementary Fig. 19l). Alizarin red staining revealed a marked increase in calcification after 14 days of OM culture (Supplementary Fig. 19m).

### Single-cell regulatory network inference and clustering analysis identified crucial regulators of MMT

To examine the transcription factors (TFs) contributing to MDSCs cell fate determination, we applied single-cell regulatory network inference and clustering (SCENIC) analysis. Top 10 regulons of each subcluster were demonstrated as a heatmap (Fig. 7d). Expression levels of TFs generated by Monocle2 were used to verify whether the individual TFs had branched expression trends (upregulated in MMT and downregulated in classical M1 polarization) (Fig. 7e). Among the 10 regulons inferred in MDSCs, there were 3 TFs (ZNF853, GATA5, MKX) hardly detected in transcriptome, and another 3 TFs (GATA4, IRF6 and SRF) did not have branched expression trends. The rest TFs (AR, FOXC1, NFIB and SOX9) were more likely to be essential regulators involved in MMT.

RT-qPCR was utilized to analyze the upregulation of specific TFs following OM induction (Fig. 7f). SOX9 was scarcely detectable due to its low expression, while AR and NFIB exhibited no time-dependent changes in expression levels. In contrast, FOXC1 levels gradually increased with prolonged culture time, indicating that FOXC1 may play a crucial role in regulating MMT. The protein level of FOXC1 also increased following OM induction (Fig. 7g), consistent with the observed trend in mRNA levels.

Besides, GSEA analysis of KEGG pathways indicated that PI3K-AKT signaling pathway was activated in MDSC (Fig. 7h, Supplementary Fig. 13). Then WB results showed that phosphorylation levels of AKT in MRC1^+^Mac upregulated after 7 days induction in OM (Fig. 7i, Supplementary Fig. 20a). Studies have shown that forkhead box family proteins [56] are related with PI3K-AKT signaling pathway, and FOXC1 might be an upstream regulator of AKT phosphorylation [57, 58]. We used small interfering RNA (siRNA) of FOXC1 to silencing its expression. Results showed that FOXC1 was significantly knocked down both at mRNA and protein levels (Supplementary Fig. 20b-c). Western blots suggested that phosphorylation level of AKT was downregulated, and expression levels of myofibro/osteoblastic markers (α-SMA, RUNX2 and Osteopotin) decreased after silencing of FOXC1 (Fig. 7j). Alizarin red staining detected a significant reduction of calcification in si-FOXC1 group (Fig. 7k-l). These results suggested that silencing of FOXC1 suppressed PI3K-AKT pathway and rescued MMT in vitro.

## Discussion

CAVD was once thought to be a passive degenerative disease, but more studies now support that it is an active process driven by interactions among resident cell populations and infiltrating immune cell populations. Underlying mechanisms could be complicated including abnormal lipid metabolism, oxidative stress, inflammatory response, ECM remodeling [44]. Obtaining a detailed cell atlas and correctly identifying the heterogeneity of valve cells is a prerequisite for further exploring potential mechanisms. Based on this, we can further investigate cell developmental trajectories and cellular interactions at the single-cell level. Currently, research has preliminarily revealed that the interaction between the resident cell population and infiltrating immnue cell population [9, 59] in the valve can promote the osteogenic differentiation of VICs, leading to valve leaflet calcification, thickening, and dysfunction [44]. However, the heterogeneity of aortic valve cells needs to be further elucidated.

Our study addresses two major issues. Firstly, as an important etiology of aortic valve calcification, BAV deserves further investigation. Due to the developmental abnormality during valve formation, the cell composition of BAV and TAV is likely to differ. We have revealed the cell atlas of BAV, filling this gap. Secondly, previous single-cell sequencing studies did not take calcification levels into consideration, making it difficult to obtain comprehensive cellular composition information of late-stage valve calcification. Xu et al. utilized scRNA-seq to provide evidence for the involvement of EndMT in the pathogenesis of CAVD [9]. They did not provide calcification score data of specimens, and based on the figure in their paper, the specimens did not show significant thickening and calcification. Moreover, their study mainly focused on EndMT and did not perform detailed clustering or further analysis of immune cells [9]. Lee et al. performed single-cell sequencing on mouse models, revealing the cellular heterogeneity of aortic valve calcification in mice [12]. They used a hyperlipidemia model (ApoE^-/-^ and Ldlr^-/-^ mice with western diet) to induce CAVD. This model typically does not result in high aortic valve blood velocity and is often utilized to study the early stages of calcification. Therefore, their research focused on the immunomodulation during hyperlipidemia. In our research, specimens with different levels of calcification were used to obtain a more complete picture of calcified valves.

We identified many cell types in BAV that were not previously recognized. In the stromal cells, we identified VIC subtypes directly involved in inflammatory activities. Additionally, we proposed the existence of pericytes in BAV, which is consistent with a previous in vitro study [42]. As to the immune cells, we identified mast cells and clustered lymphocytes in BAV, which provides a detailed cell atlas for future research. Based on RNA velocity and trajectory analyses, we explored monocytic lineage in BAV, provided more evidence that macrophages can switch to a pro-inflammatory state in calcified BAV.

More importantly, we discovered the presence of MDSC phenotype and potential mechanism of MMT which might be involved in bicuspid CAVD. Although macrophages in valves expressing stromal markers have been reported previously, they did not delve into their origin and function. Shu et al. collected non-calcified cardiac valves from end-stage heart failure patients to conduct scRNA-seq analysis [45]. Interestingly, a subpopulation of macrophages expressing the mesenchymal markers MGP, COL1A2, and DCN was also found in this study, which the researchers named as *remodeling macrophages* [45]. This subpopulation is very similar to the MDSCs in our study. Although osteogenic markers were not significantly elevated in the *remodeling macrophages*, it is possible that this result was due to the fact that only non-calcified valves were included in the study [45]. While trajectory analysis was not performed on the macrophage lineage by Shu et al. [45], we could tentatively infer that this subpopulation might have similarities in origin to the MDSCs in our research.

The term MMT was first introduced by Nikolic-Paterson et al. to describe the conversion of monocytes to myofibroblasts in renal fibrosis [60]. Since then, Wang et al. have demonstrated, through lineage-tracing mice, that macrophages can exacerbate renal fibrosis by developing MMT [20]. Moreover, MMT has also been reported in studies of pulmonary fibrosis [61] and pancreatitis [22]. However, MMT has received limited attention in cardiovascular disease research. Haider et al. reported that macrophages can convert into fibroblast-like phenotypes after myocardial infarction, playing a role in post-infarction repair [21]. In the context of CAVD, Oba et al. found that many M2 macrophages (MRC1^+^Mac) co-expressed the osteogenic marker BMP2 through histological studies [48]. Nevertheless, there is insufficient evidence to prove that macrophages are one of the sources of OBLCs in the calcific aortic valves. Through a combination of scRNA-seq analysis, histopathological assays, and in vitro validation, we provided evidence for MMT involvement in BAV calcification and further characterize the phenotype of MDSCs.

The role of MRC1^+^Mac in the progression of CAVD has been underappreciated. Recent studies have revealed that TGF-β released by MRC1^+^Mac can promote the osteogenic transdifferentiation of VICs [62, 63]. Our results showed that the number of MRC1^+^Mac increased with aggravation of valvular calcification. Therefore, we assumed that the role MRC1^+^Mac played in CAVD progression could not be neglected. In our study, by applying RNA velocity and trajectory analysis techniques, we found that MRC1^+^Mac in the calcific valves obtained a tendency to shift towards a pro-inflammatory state, and also had the potential to transdifferentiate into myofibro/osteoblasts, consistent with previous studies on MMT related to kidney and lung [61, 64, 65]. Recent studies also have reported that osteoclast-like cells (OCLCs) existed in calcific aortic valves [66, 67], however the origin of these cells was unclear. In atherosclerotic plaques, OCLCs with impaired capacity of mineral uptake derived from M2 macrophages was observed around calcific deposits [68]. Although we did not identify a distinct population of osteoclasts via scRNA-seq, we could still make an assumption that MRC1^+^Mac, especially LYVE1^hi^MRC1^+^Mac, probably be a main source of OCLCs in calcific aortic valves for the expression of CTSK and ACP5 (TRAP). Collectively, we proposed that MRC1^+^Mac could directly remodel the ECM, and the phenotype of MDSC could be one of the sources of myofibroblasts, OBLCs and OCLCs in calcific aortic valves.

Several limitations should be acknowledged in our study. Firstly, since no normal BAV sample was obtained, we used stenotic aortic valve specimen with no calcification instead of non-diseased valves as control. Secondly, scRNA-seq of calcified BAV specimens is challenging (Supplementary Video 2), and therefore we used a relatively small sample size for single-cell sequencing. If we could detect more biological replicates, our conclusions would be more reliable. Thirdly, many cell types have not been histologically validated, and more work need to be conducted for further research. Although we did not identify a MDSC population in TAV dataset, it does not necessarily indicate that this phenotype does not exist in TAV calcification. The outdated reagents and instruments used for sequencing in the previous dataset may have resulted in some transcripts not being detected due to insufficient sequencing depth. Therefore, further research and validation are needed for the MMT phenotype.

## Conclusions

In conclusion, with an unbiased scRNA-seq approach, we provide a full spectrum of cell populations in stenotic BAVs. Based on the cell atlas we identified, a cellular interaction network was constructed. In particular, we identified a previously unrecognized calcification associated MDSC population and explored the mechanism of MMT. The detailed scRNA-seq information can also serve as a rich resource for further studies of CAVD.

## Electronic supplementary material

Below is the link to the electronic supplementary material.

Supplementary Material 1

Supplementary Material 2

## Data Availability

All data used for the current study are available from the corresponding author (zhudanmd@163.com) upon reasonable request. Raw data of scRNA-seq will be uploaded to GEO database before acceptance of this manuscript.

## List of abbreviations

CAVD: Calcific Aortic Valve Disease
BAV: Bicuspid Aortic Valve
VIC: Valvular Interstitial Cell
ECM: Extracellular Matrix
scRNA-seq: single-cell RNA sequencing
UMI: Unique Molecular Identifier
MDSC: Macrophage Derived Stromal Cells
MMT: Macrophage-to-Mesenchymal Transition
UMAP: Uniform Manifold Approximation and Projection
GSEA: Gene Set Enrichment Analysis
TFs: Transcription Factors
ILH: Intraleaflet Haemorrhage
OBLC: Osteoblast-Like Cells
GM: Grow Medium
OM: Osteogenic Medium
siRNA: small interfering RNA
