# Supplementary material for: Single-cell transcriptomics reveals cellular heterogeneity and macrophage-to-mesenchymal transition in bicuspid calcific aortic valve disease
Source: Biol Direct. 2023 Jun 30;18:35. doi: 10.1186/s13062-023-00390-w (PMC10311753; doi:10.1186/s13062-023-00390-w)
Supplement: Supplementary file 1 — Supplementary Material 1 [file 13062_2023_390_MOESM1_ESM.docx]

Single-Cell Transcriptomics Reveals Cellular Heterogeneity and Macrophage-to-Mesenchymal Transition in Bicuspid Calcific Aortic Valve Disease

Tao Lyu^1†^, Yang Liu^2†^, Binglin Li^1^, Ran Xu^3^, Jianghong Guo^4*^, Dan Zhu^1*^

^*^Co-corresponding authors: Tel: +86 13671573807, Email: zhudanmd@163.com (D.Z.); Tel: +86 18800351921, Email: vamondlt@outlook.com (J.G.)

## Supplementary Materials

## Supplementary Figures

**
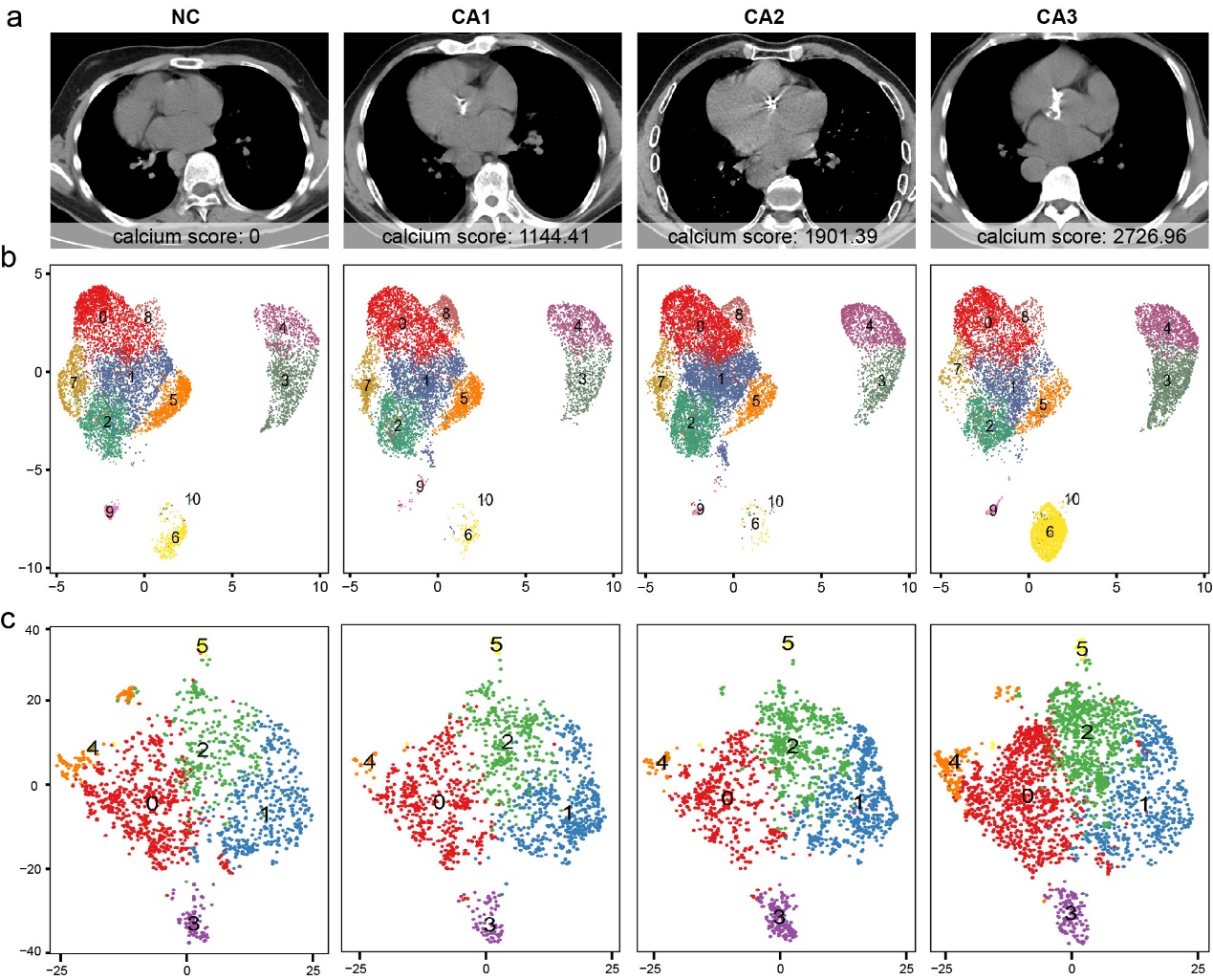
**

**Supplementary Figure 1** CT scans and cellular constitution of each specimen. **(a)** CT images and calcium score calculated by IntelliSpace Portal (Philips). **(b)** UMAP projection of all cells split by specimen. **(c)** tSNE projection of all cells split by specimen.

**
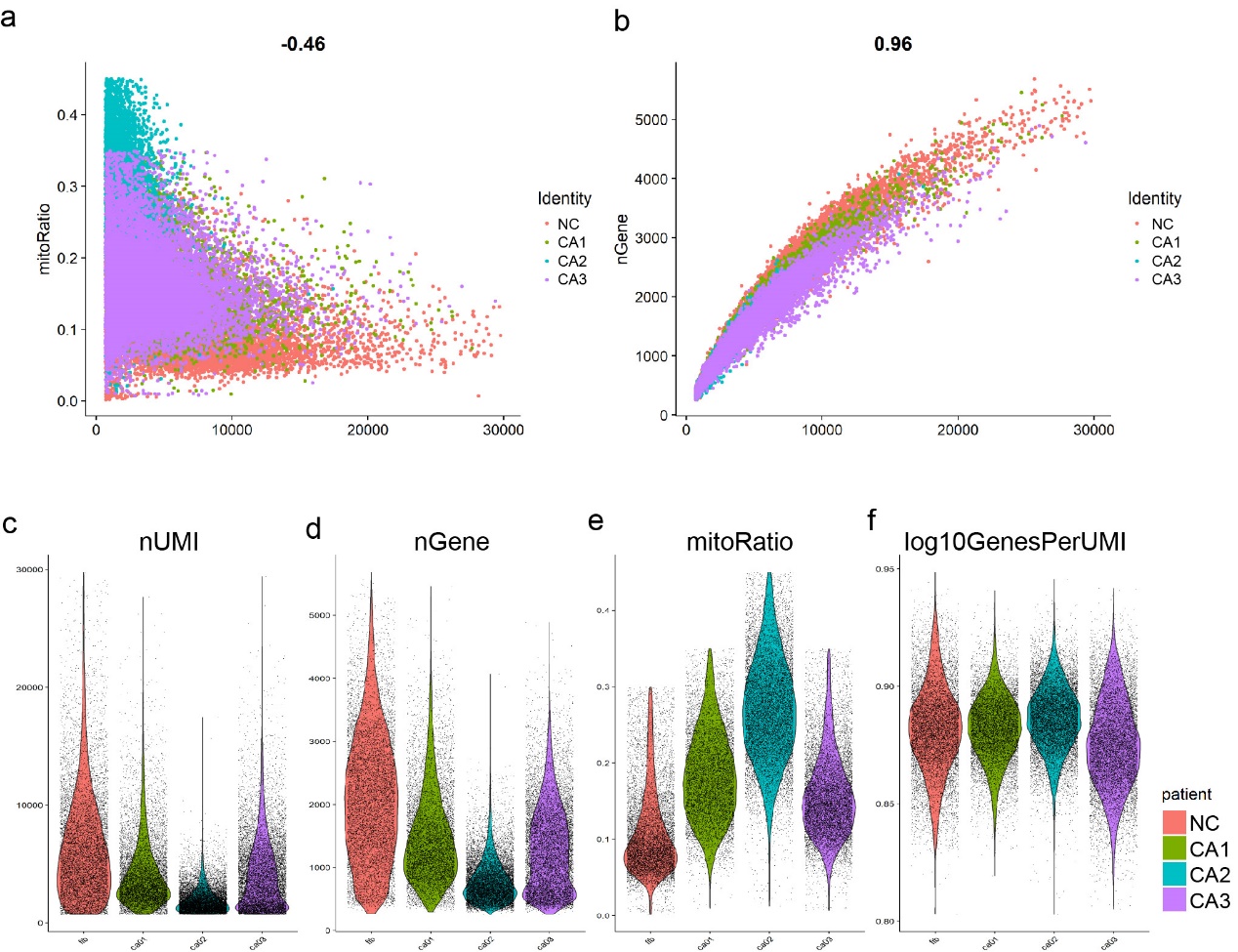
**

**Supplementary Figure 2** Quality control of scRNA data show appropriate strategy. **(a)** mitoRatio did not correlated with UMI. **(b)** Number of genes positively correlated with UMI. **(c)- (f)** Violin plots of nMUI (c), nGene (d), mitoRatio (e), log10GenesPerUMI(f) after Quality control.


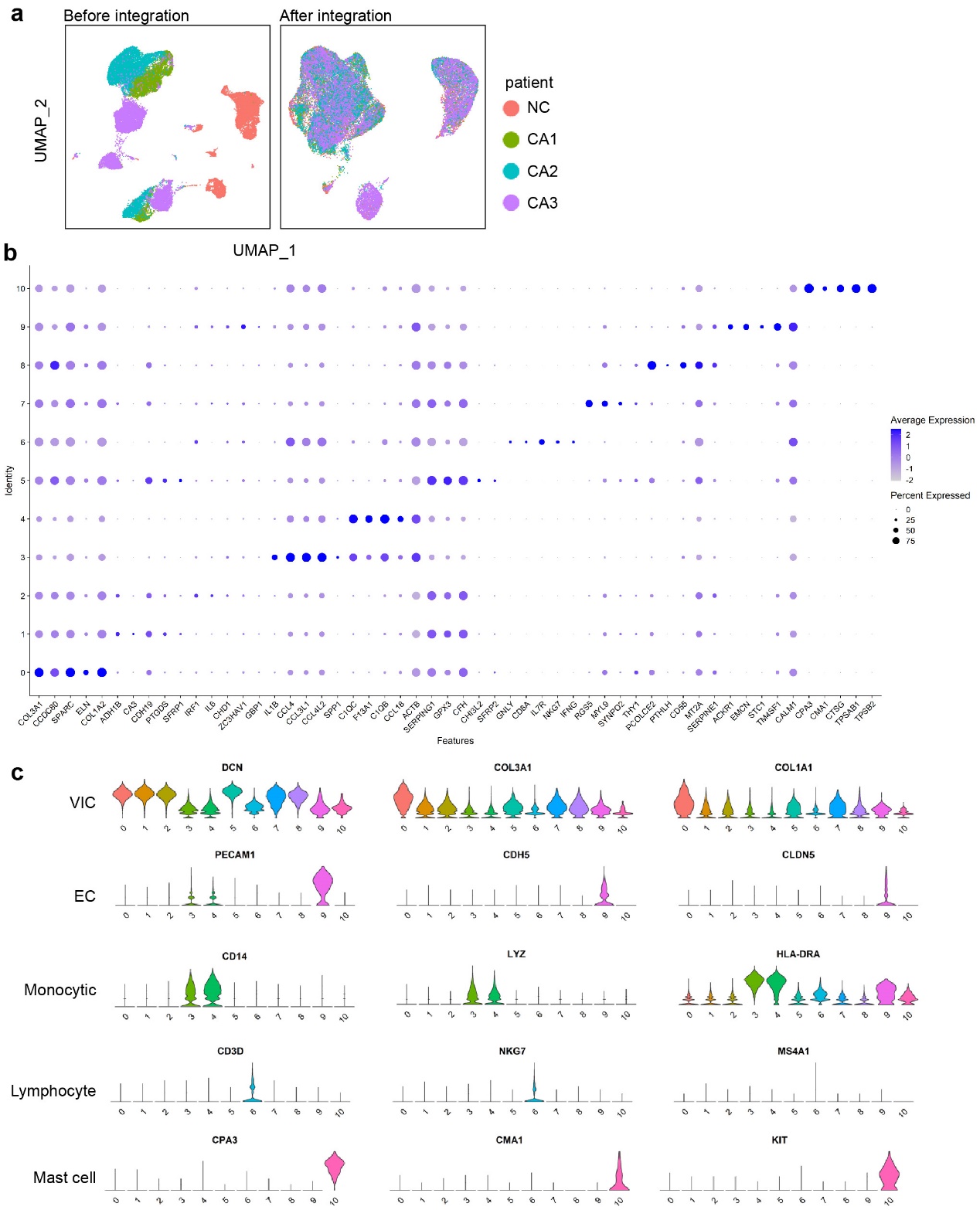


**Supplementary Figure 3** Major clusters and markers of scRNA-seq data. **(a)** UMAP projection before and after integration procedures. **(b)** Distribution of major cell types in different specimens. **(c)- (d)** Feature plots (c) and violin plots (d) of selected markers for cell types.


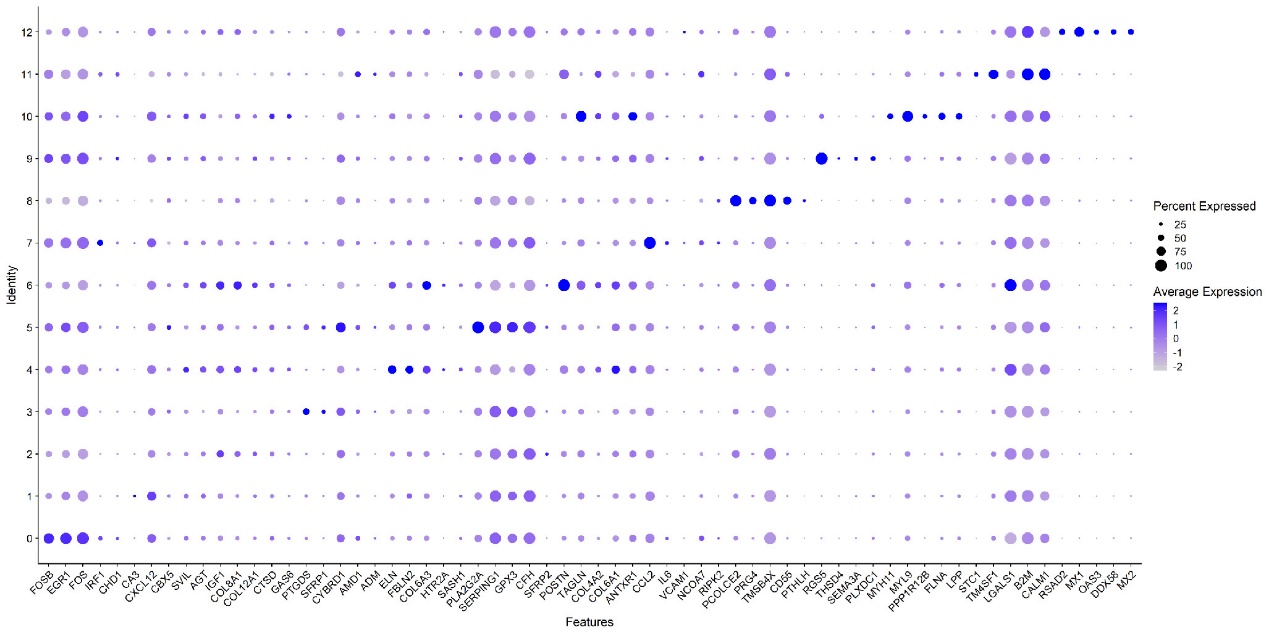


**Supplementary Figure 4** Top 5 marker genes for subclusters of sromal cells generated by Seurat *FindMarkers* function.


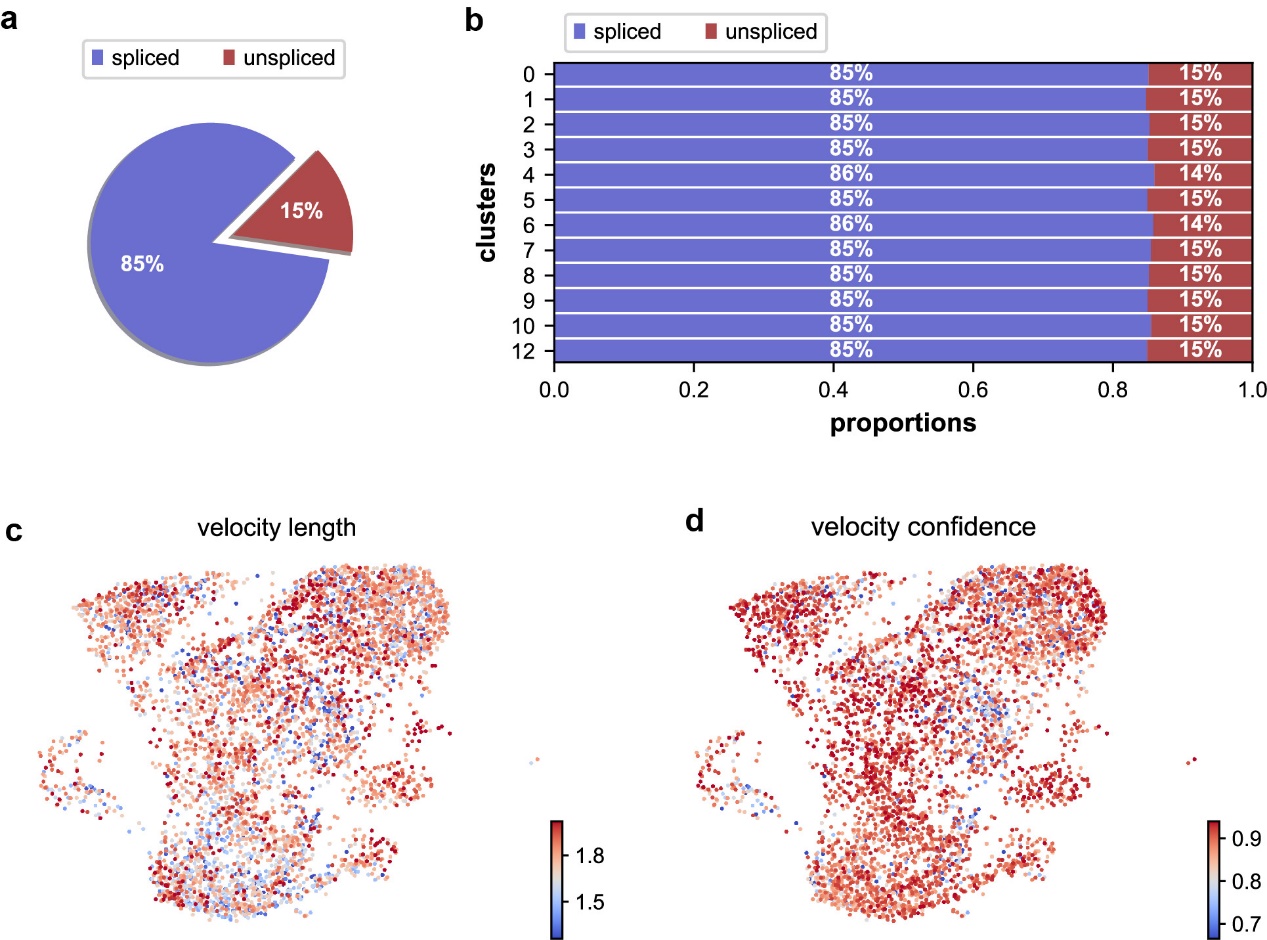


**Supplementary Figure 5** Supplementary images for RNA velocity and trajectory analyses. **(a)- (b)** proportion of spliced and unspliced RNA in total (a) and in each subcluster of stromal cells (b). **(c)- (d)**velocity length (c) and velocity confidence (d) calculated by scVelo.


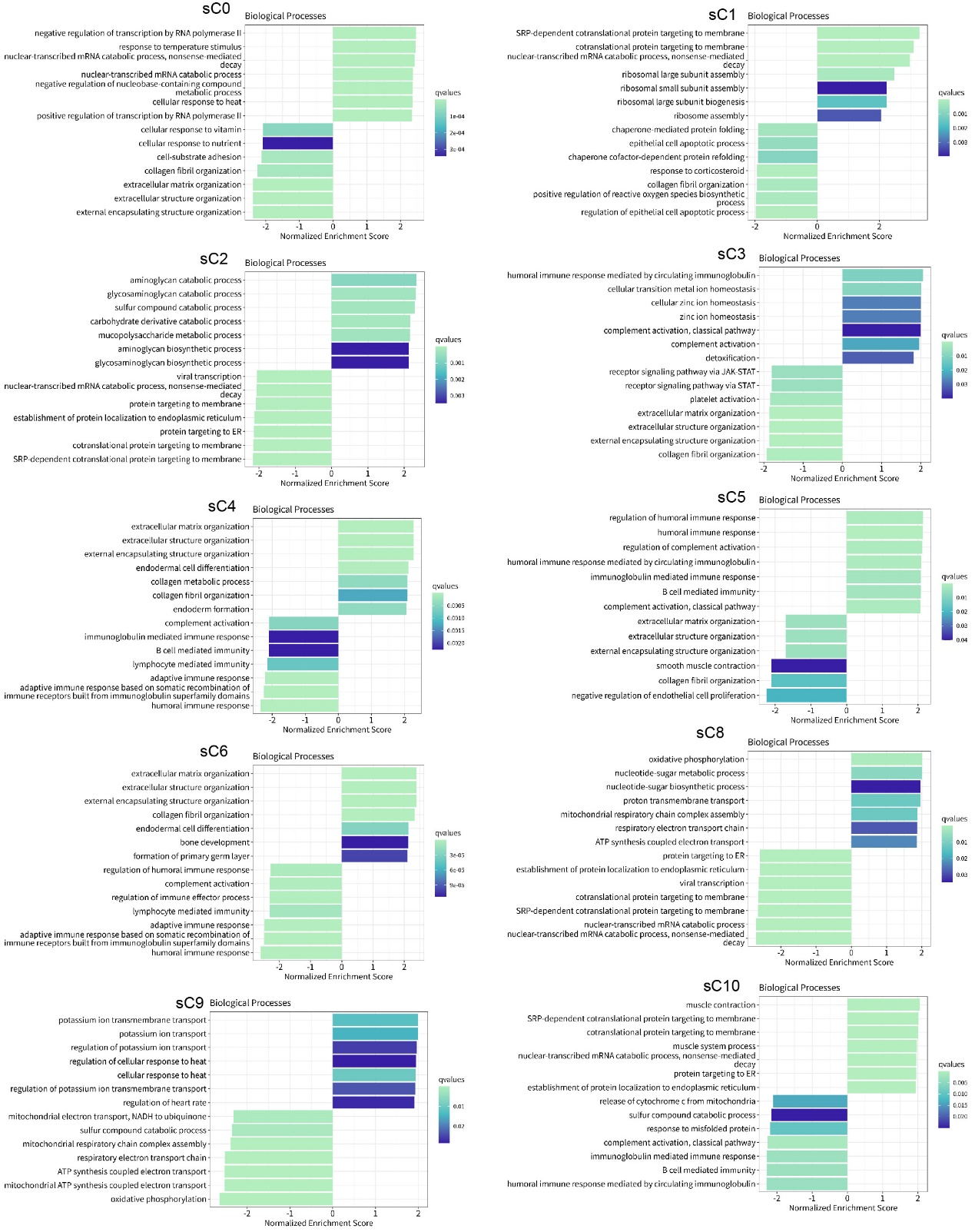


**Supplementary Figure 6** GSEA analyses of biological processes in stromal cells.


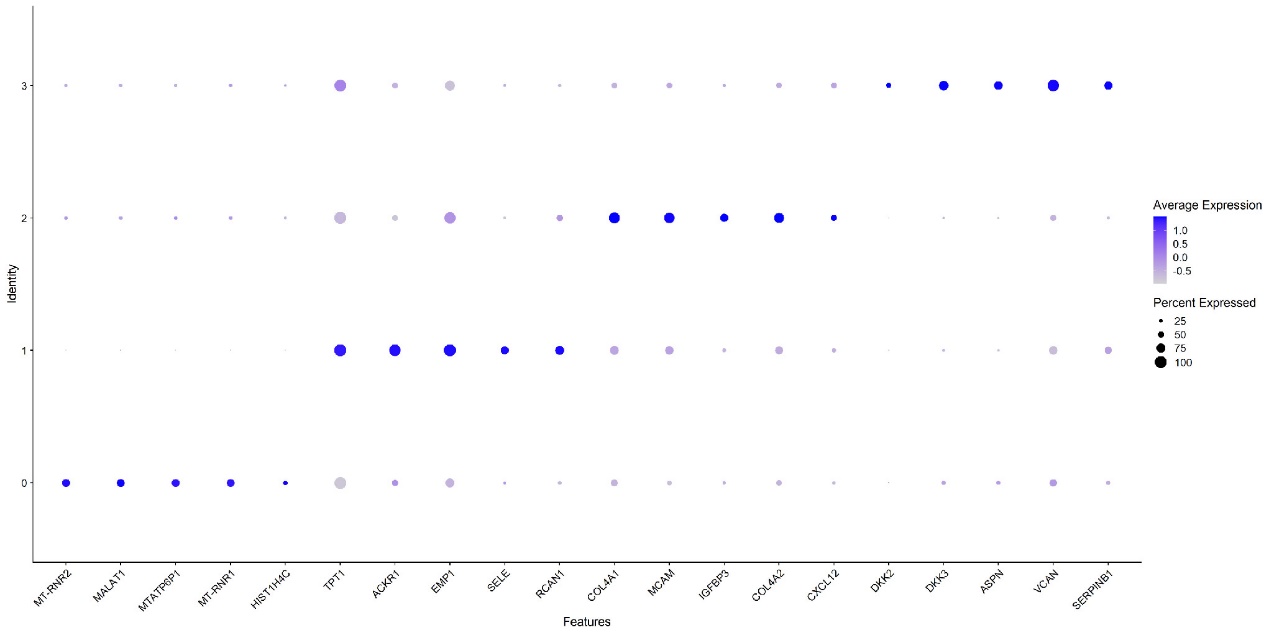


**Supplementary Figure 7** Top 5 marker genes for subclusters of ECs generated by Seurat *FindMarkers* function.


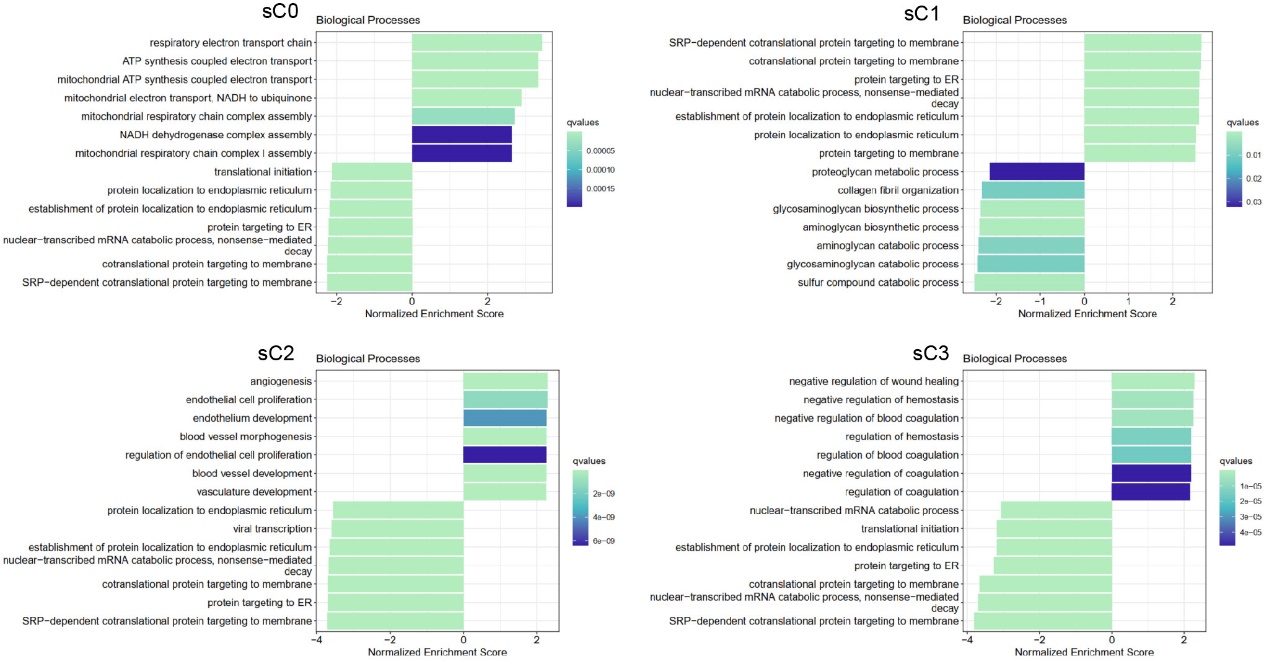


**Supplementary Figure 8** GSEA analyses of biological processes in ECs.


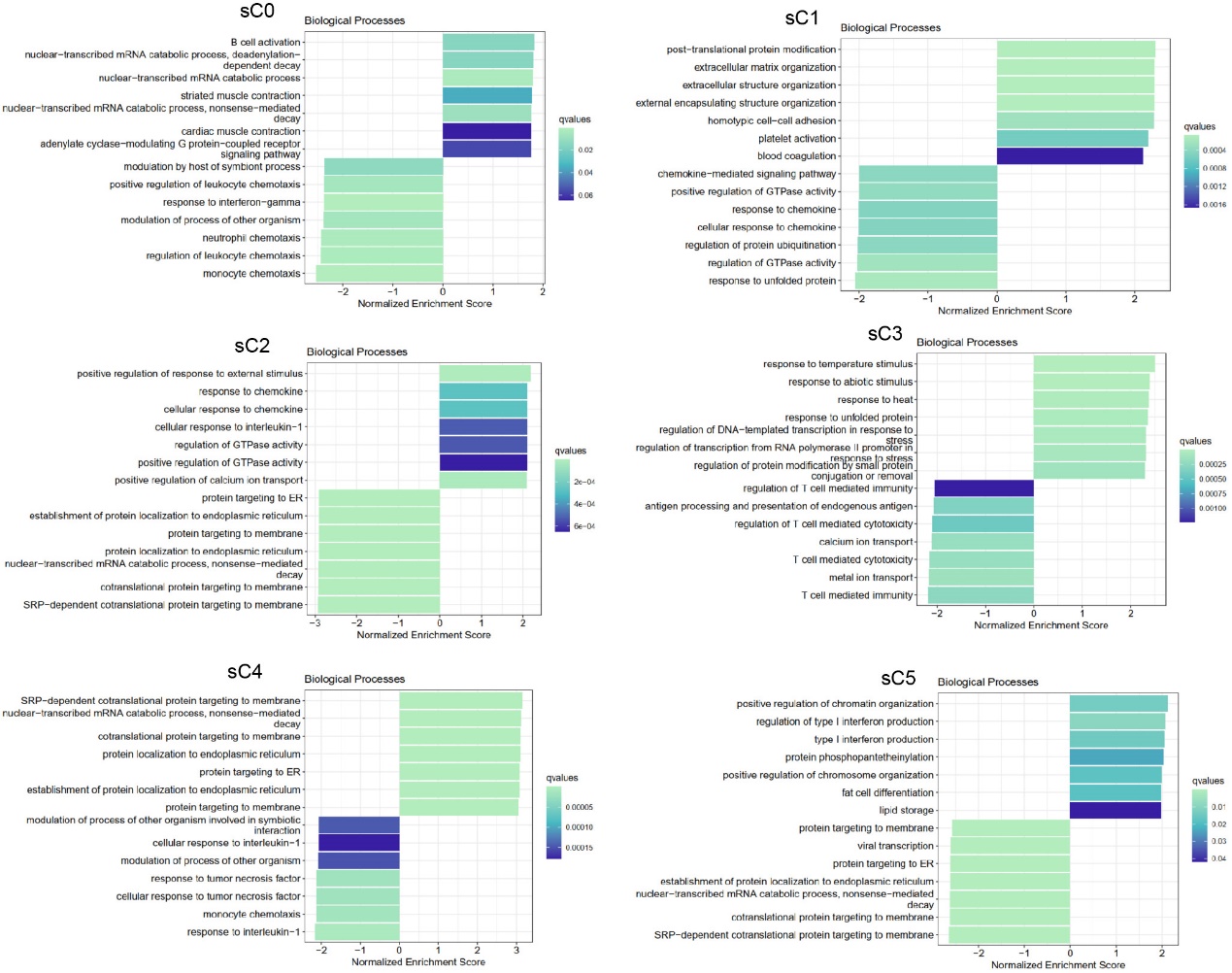


**Supplementary Figure 9** GSEA analyses of biological processes in lymphocytes.


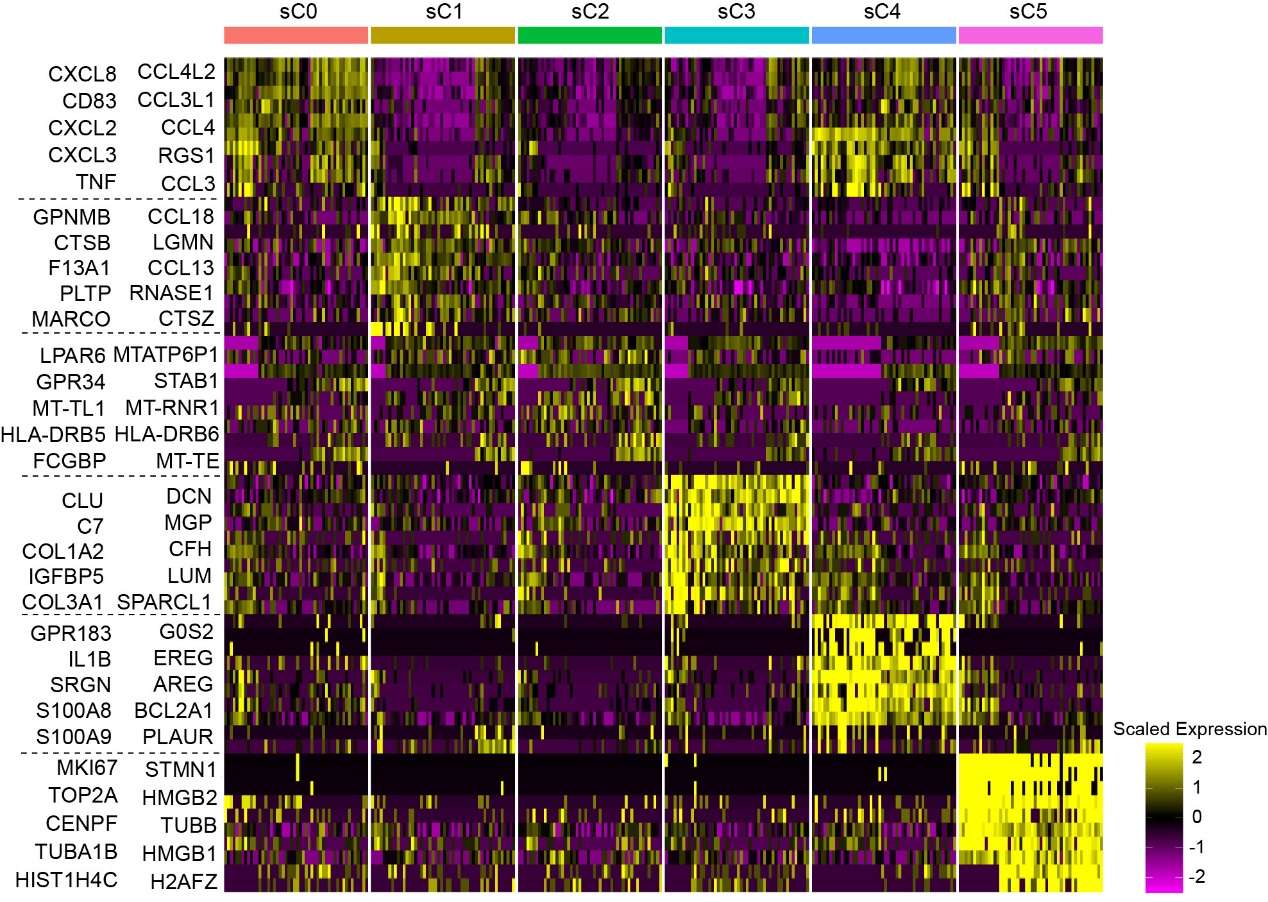


**Supplementary Figure 10** Top 10 marker genes for subclusters of monocytic cells generated by Seurat *FindMarkers* function.


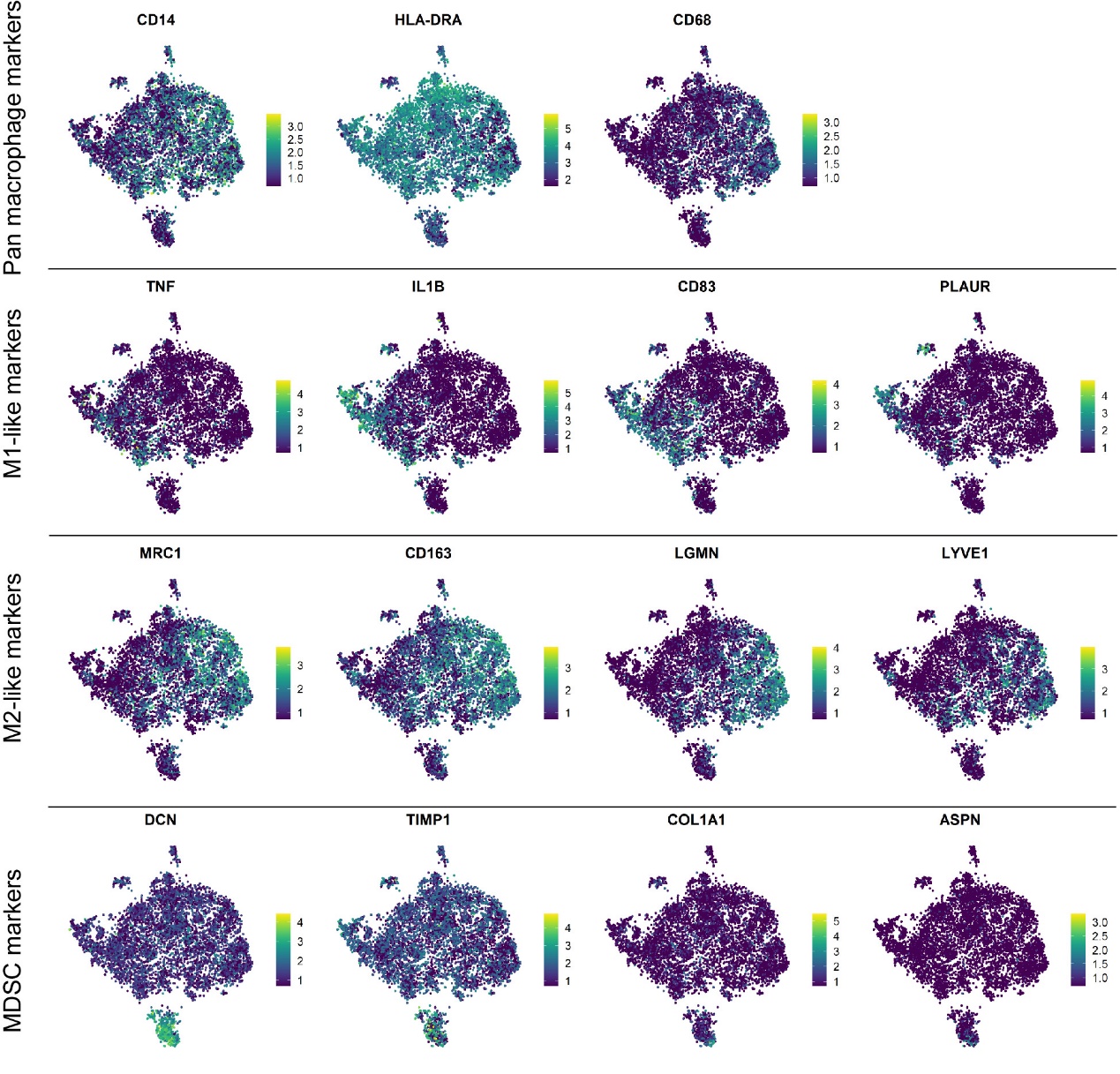


**Supplementary Figure 11** Important markers for monocytic subclusters presented as featureplot.


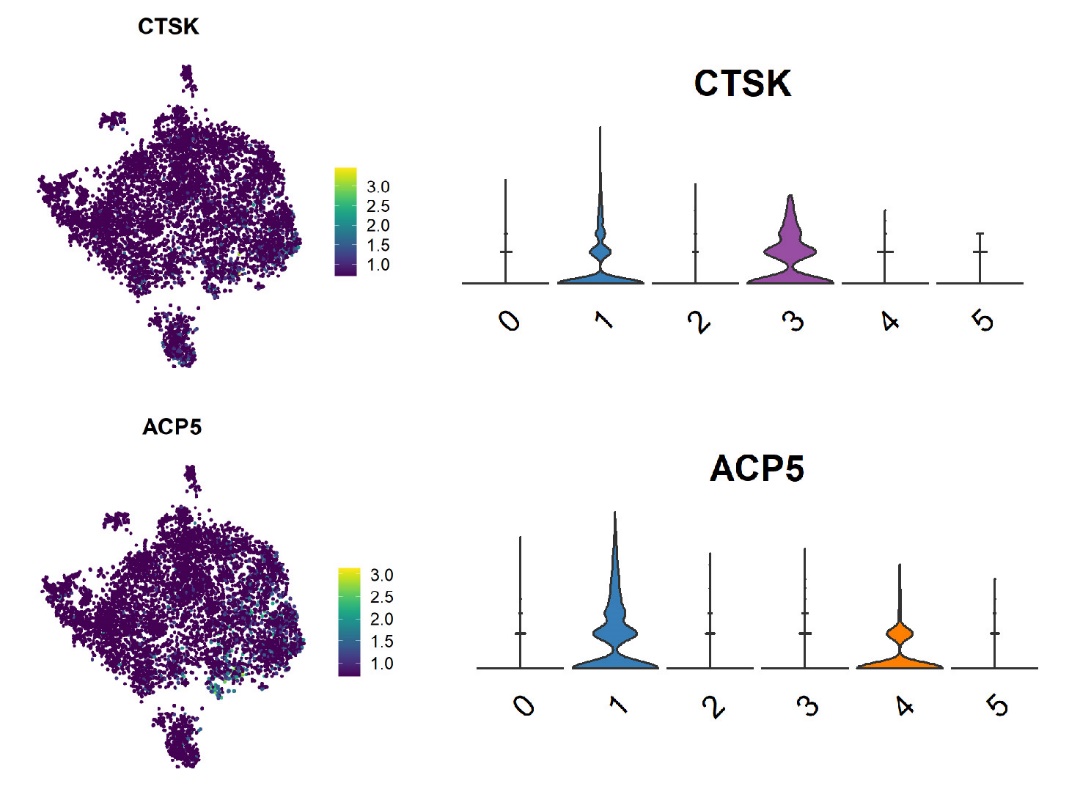


**Supplementary Figure 12** sC1 (LYVE1^+^MRC1^+^Mac) expressed both osteoclast markers (CTSK and ACP5).

**
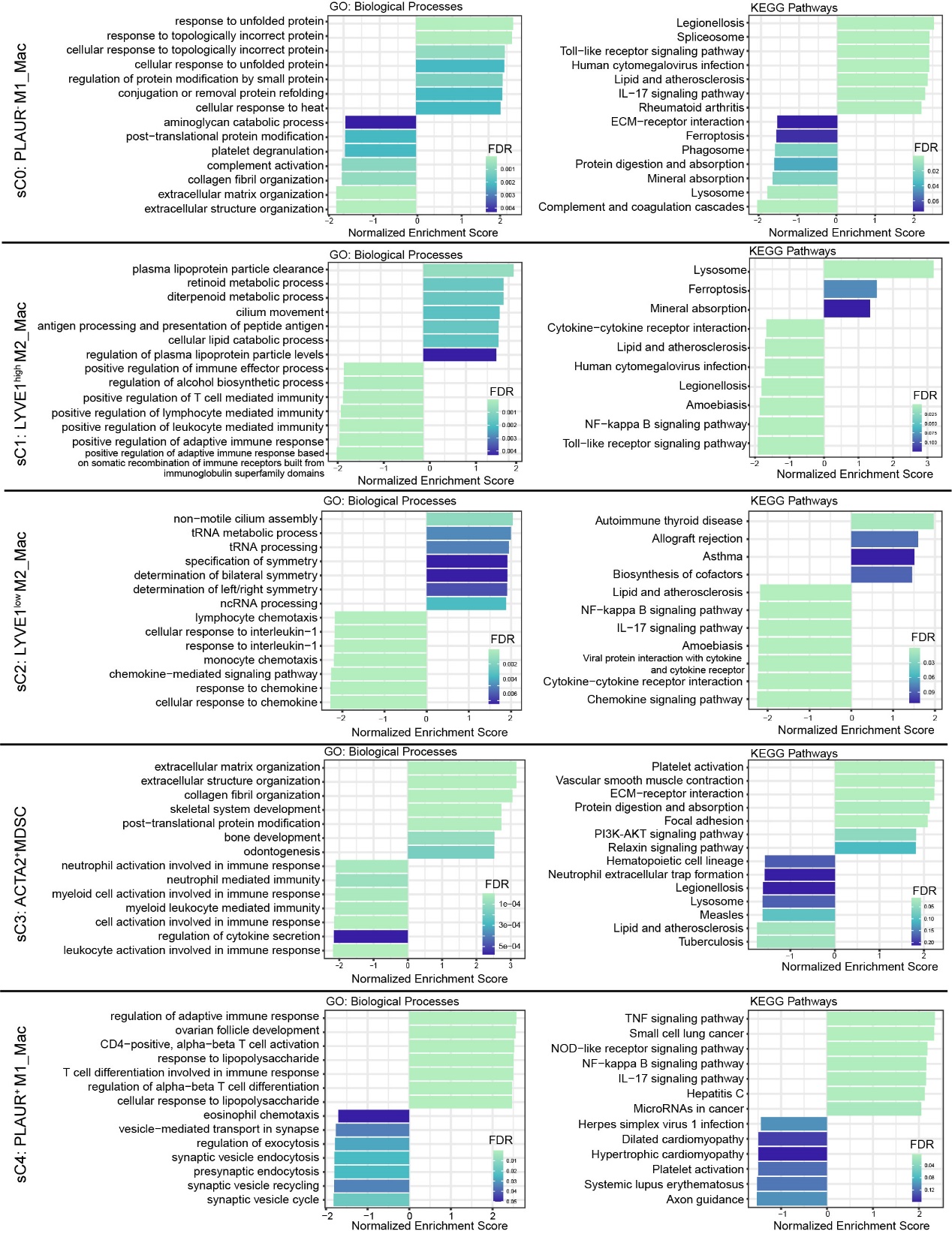
**

**Supplementary Figure 13** GESA results of monocytic subclusters using GO and KEGG databases. Only top 7 upregulated/downregulated pathways were demonstrated.


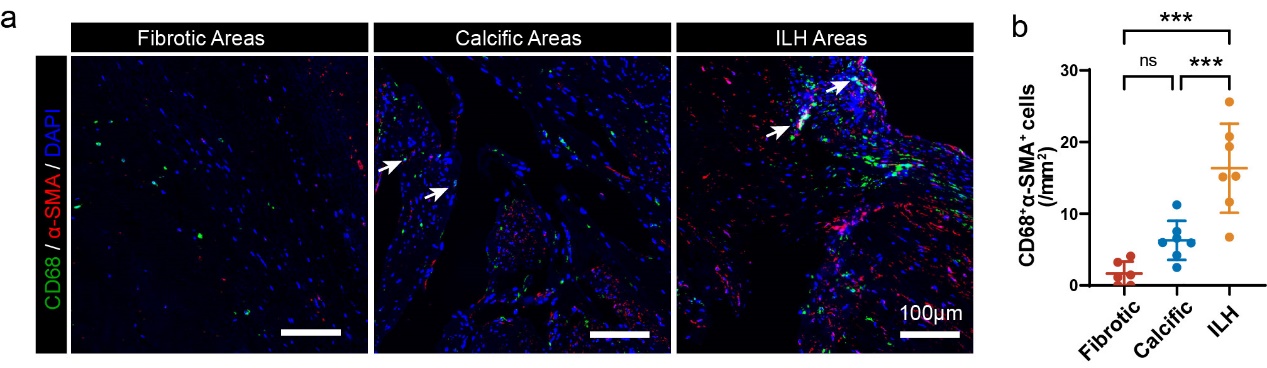


**Supplementary Figure 14** Histological analysis of CD68 and α-SMA co-expressing cells in calcified BAVs **(a)** Representative images of paraffin sections from human calcific BAV tissues (n=7). Every specimen was separated into three areas: fibrotic areas, calcific areas and ILH areas. Anti-α-SMA: red, anti-CD68: green, DAPI: bule. Scale bar: 100μm. **(b)** Quantitative results of CD68^+^α-SMA ^+^cells.


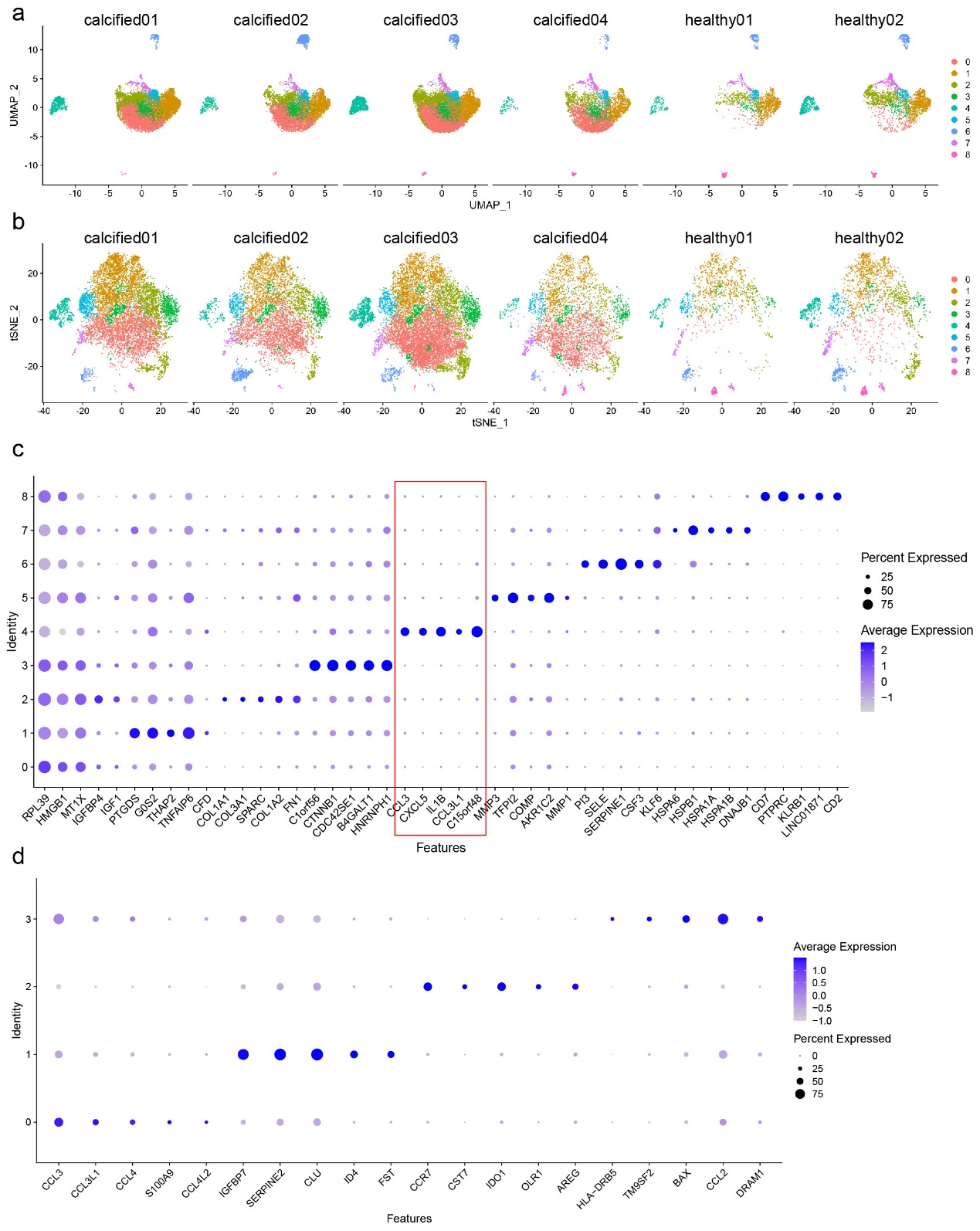


**Supplementary Figure 15** Re-analysis of previous scRNA-seq dataset of CAVD. **(a)** UMAP projection of all cell clusters split by specimen ID. **(b)** tSNE projection of all cell clusters split by specimen ID. **(c)** Top 5 markers of each cluster were presented as dotplot. Cluster4 was identified as monocytic cells. **(d)** Top 5 markers of detailed subclusters of monocytic cells were presented as dotplot.


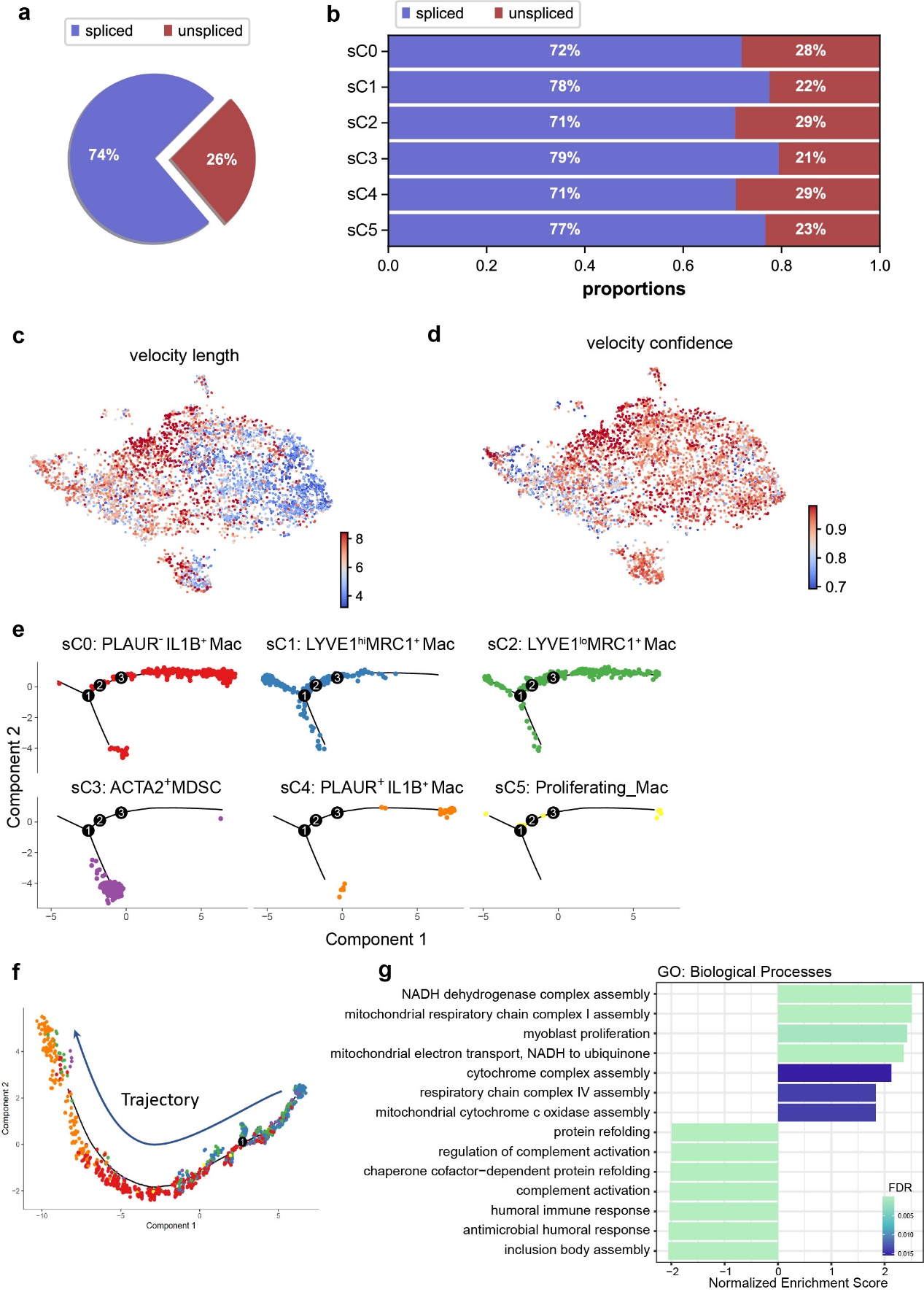


**Supplementary Figure 16** Supplementary images for RNA velocity and trajectory analyses of monocytic cells. **(a)- (b)** proportion of spliced and unspliced RNA in total. (a) and in each subcluster (b). **(c)- (d)**velocity length (c) and velocity confidence (d) calculated by scVelo. **(e)** Trajectory of non-calcific specimen (fib) built by Monocle2. **(f)** GO enrichment results using DEGs of MDSCs (calcific vs non-calcific) as input.


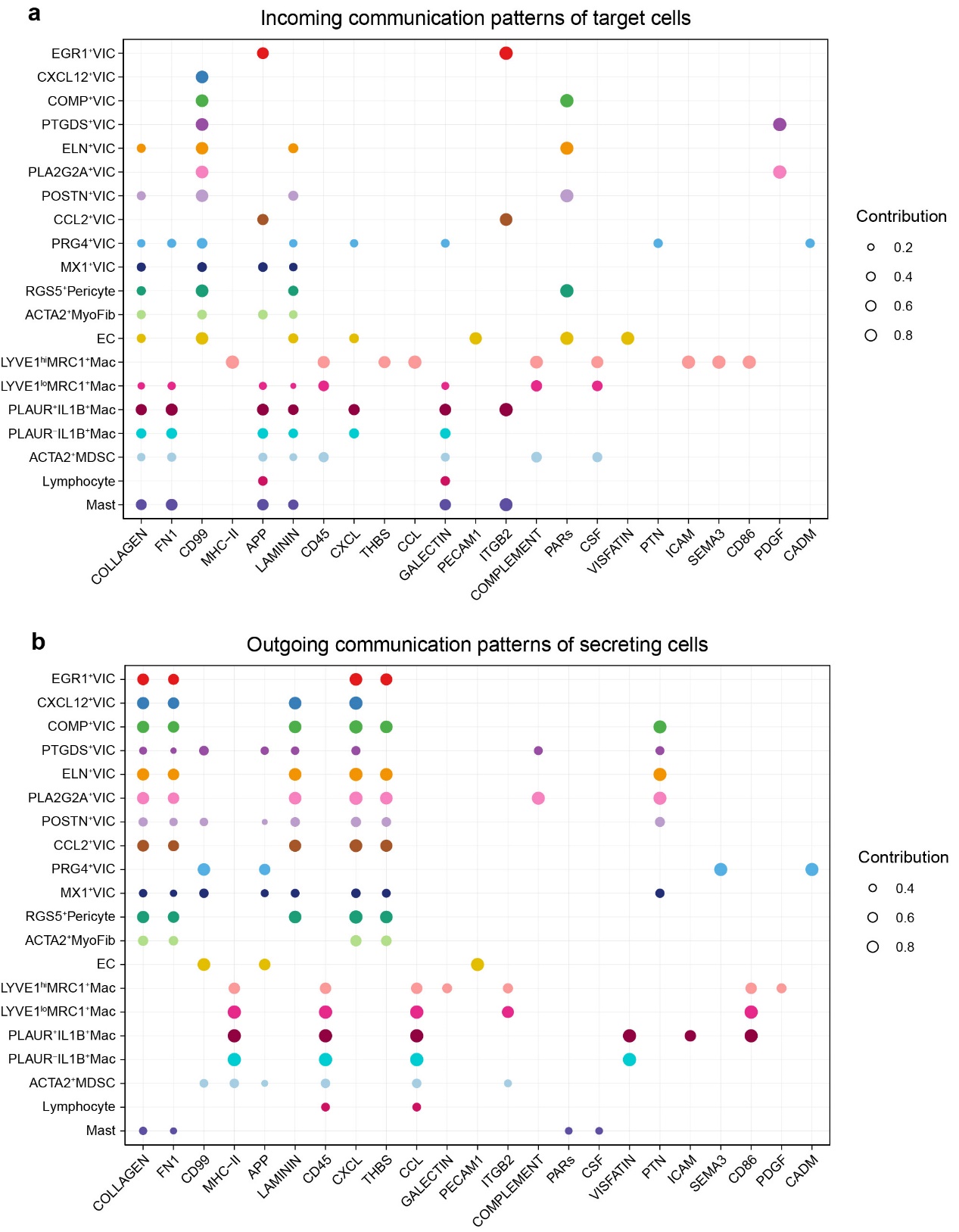


**Supplementary Figure 17** Cellular interaction network of all cell types in BAV. Represented as incoming signaling **(a)** and outgoing signaling **(b)**.


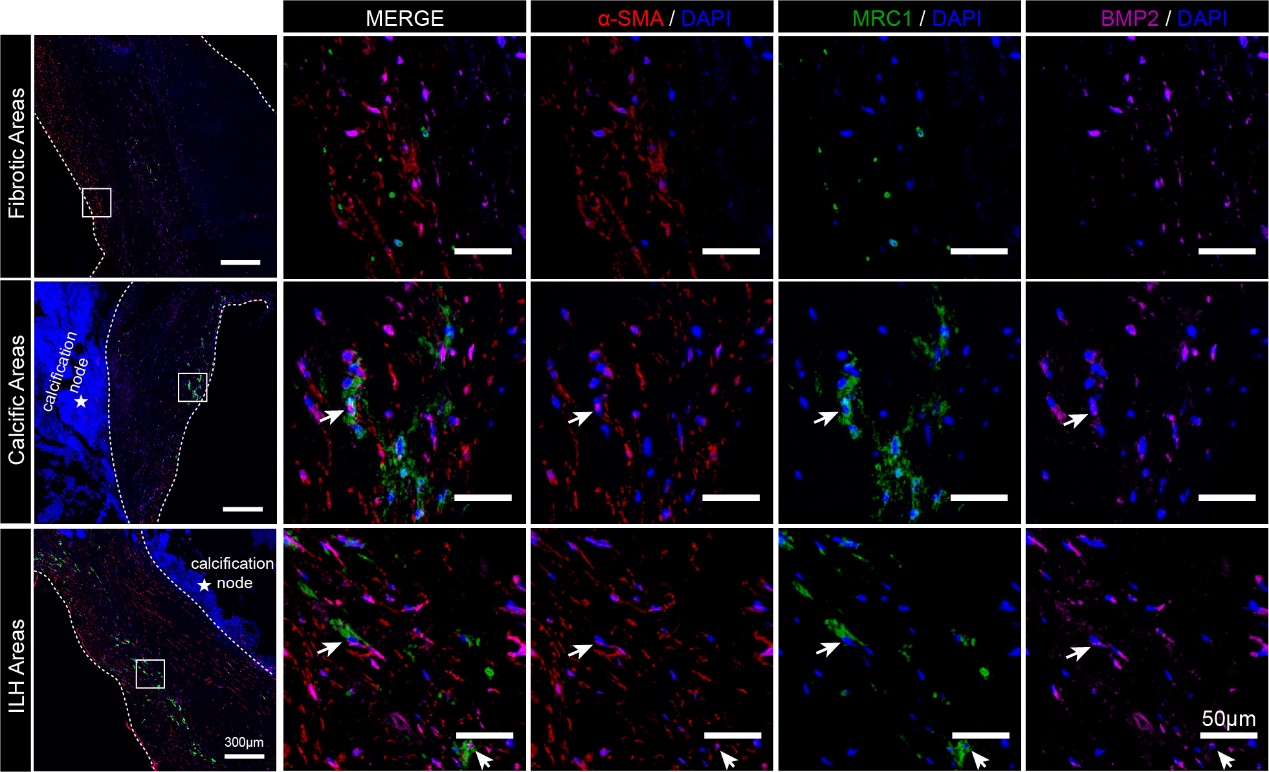


**Supplementary Figure 18** Multiplex immunohistology analysis of human calcified BAV specimens. Figure shows representative images of paraffin sections from human calcified BAV tissues (n=7). Anti-α-SMA: red, anti-MRC1: green, anti-BMP2: purple, DAPI: bule. Scale bar: 300μm. Inset scalebar: 50μm. Numbers of certain cells were manually counted, areas of sections with tissue were measured using Image J.


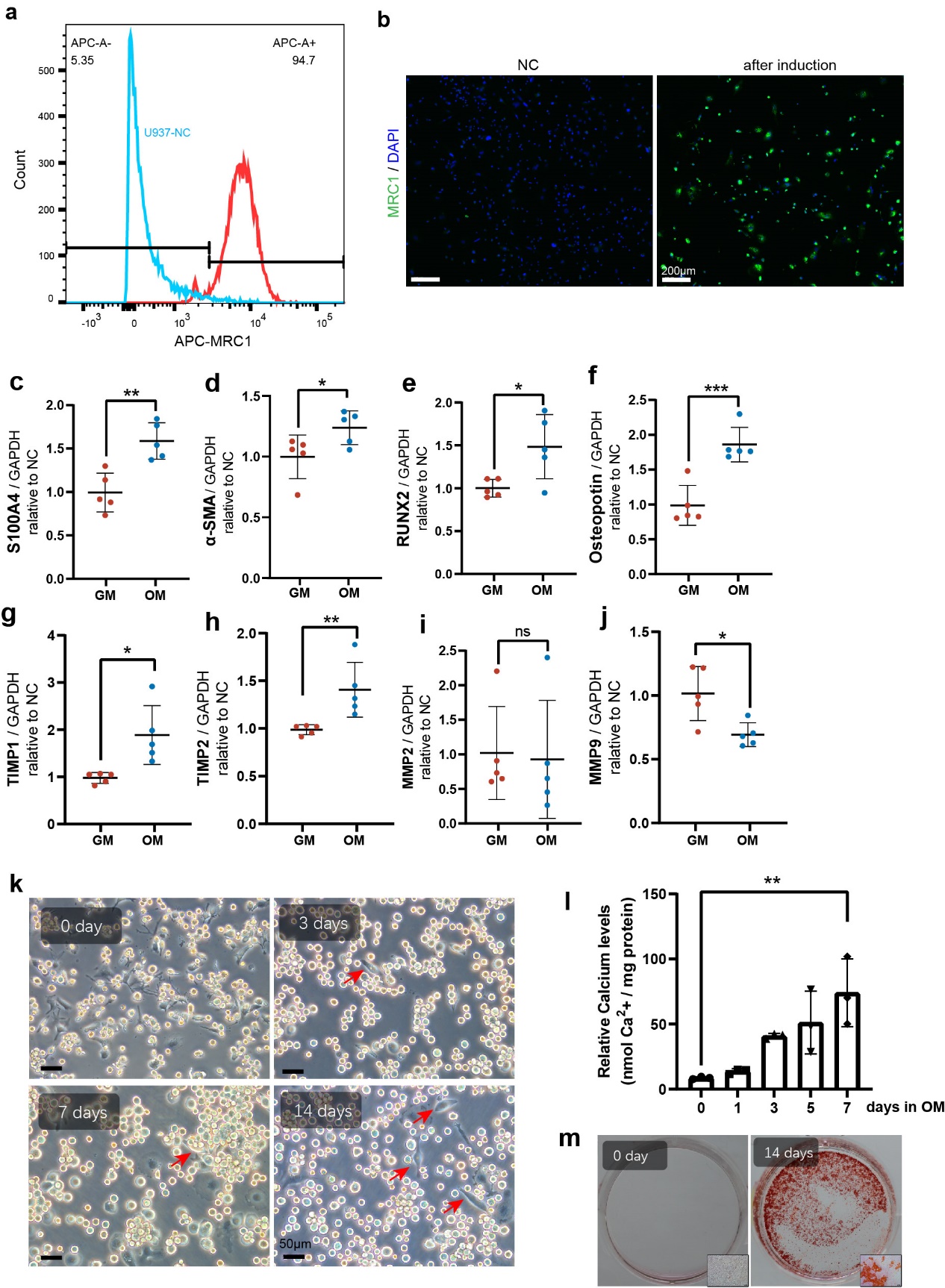


**Supplementary Figure 19** In Vitro analysis of MMT. **(a)** Flow cytometry showed about 94.7% cells expressing MRC1 (CD206) after induction. **(b)** Immunofluorescent staining showed most cells expressing MRC1 after induction. **(c-j)** Western blot results of fibro/osteoblastic markers and ECM related proteins. MRC1^+^Macs were incubated in OM for 7 days. (n=5,5 for GM, OM) Quantitative analysis of S100A4 (**c**), α-SMA (**d**), RUNX2 (**e**), Osteopotin (**f**), TIMP1 (**g**), TIMP2 (**h**), MMP2 (**i**), MMP9 (**j**). **(k)** Phase contrast images of MRC1^+^Mac after different induction time in OM. With the increase of incubation time, the shape of MRC1^+^Mac transformed from typical macrophages with pseudopodia to spindle-shaped stromal morphology. Scale Bar: 50μm. **(l)** Relative cellular calcium levels quantified by calcium measurement assay (normalized to protein levels). (n=3) **(m)** Alizarin red staining showed increased calcium deposit after 14 days induction in OM.


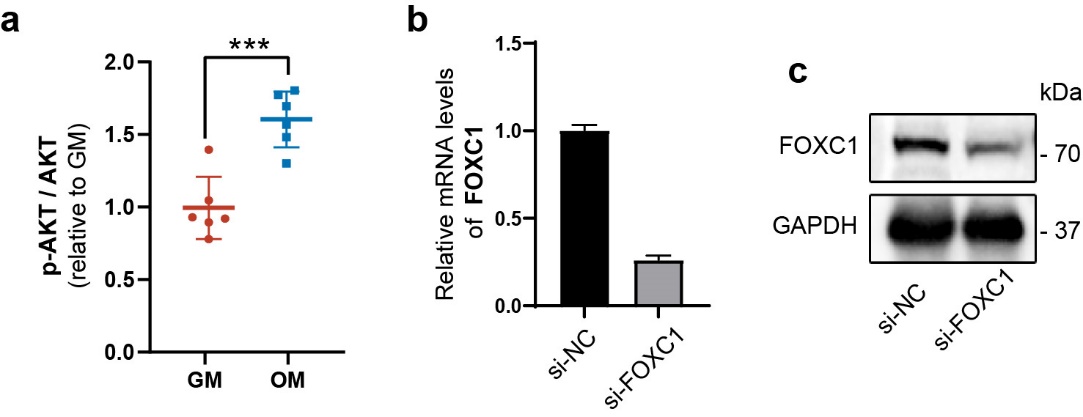


**Supplementary Figure 20** Quantitative results of p-AKT and validation of FOXC1 silencing. **(a)** The phosphorylation levels of AKT upregulated in vitro after OM induction. (n=6) **(b)-(c)** Silencing of FOXC1 in U937-induced MRC1^+^Mac. FOXC1 expression decreased both at mRNA (**b**) level and protein (**c**) level.

## Supplementary Videos


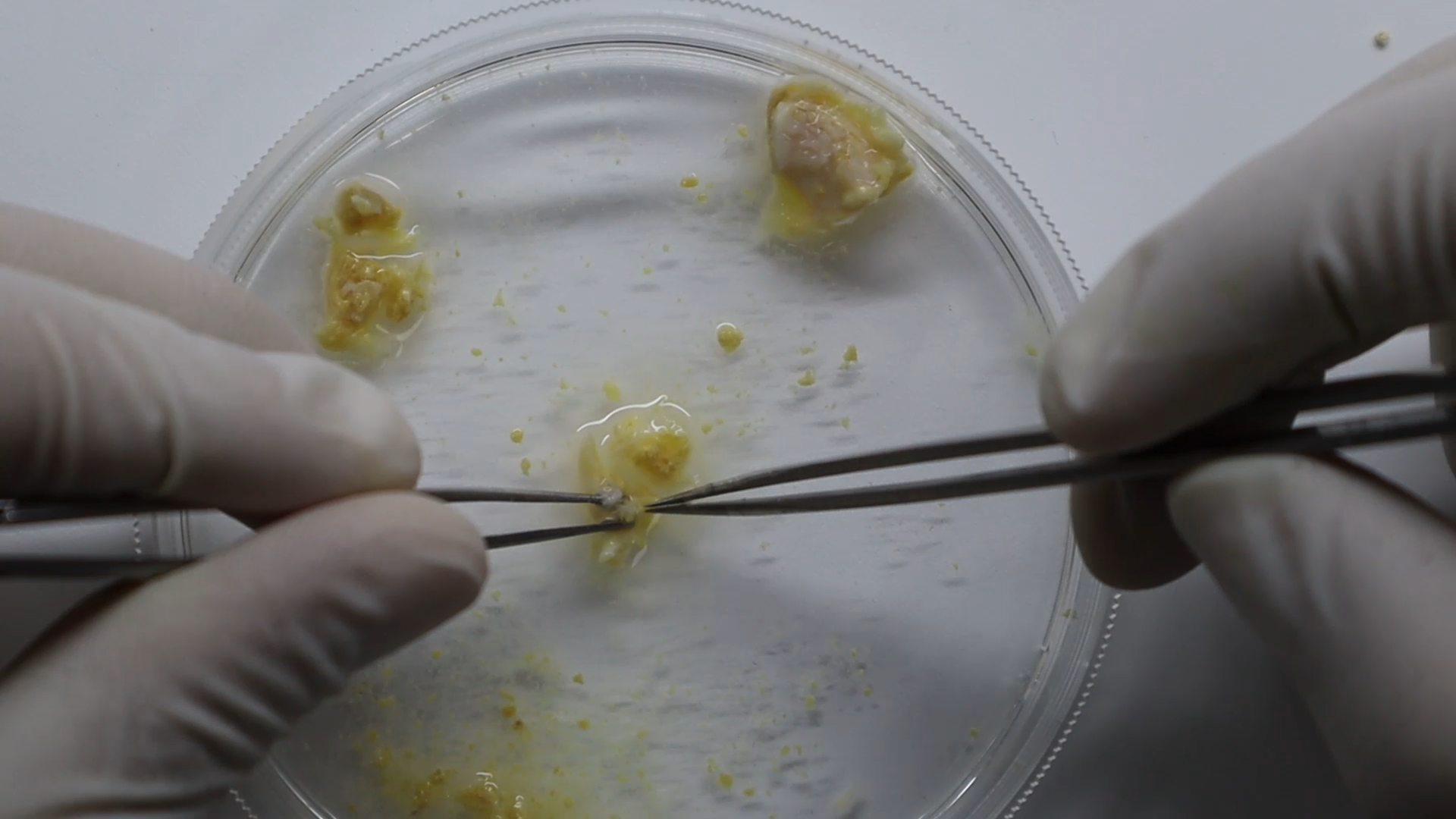


**Supplementary Video 1** Why scRNA-seq of CAVD is challenging? The video shows the process of removing large calcification nodules in a calcified valve. In our study, we attempted to obtain single-cell suspensions from 7 calcific valves, but only 3 of these specimens were suitable for sequencing, while the others did not yield enough cells or had inadequate cellular viability. One factor contributing to this limitation is the presence of high levels of calcification and necrotic cells in specimens from patients with severe aortic stenosis, which often make it challenging to obtain qualified single-cell suspensions for sequencing. Additionally, patients with mild calcific aortic valves usually present with mild stenosis, which makes them ineligible for surgical intervention.

## Supplementary Tables

Supplementary Table 1: Patients information for 4 samples of scRNA-seq

| **Patient** | **Sex** | **Age** | **CT-AVC (score)** | **Aortic valve Hemodynamics** | | | **Heart function** | | **Other risk factors** | | |
| --- | --- | --- | --- | --- | --- | --- | --- | --- | --- | --- | --- |
|  |  |  |  | **regurgitation** | **Jet velocity (m/s)** | **Mean Pressure gradient (mmHg)** | **LVEF (%)** | **NYHA Class** | **Diabetes** | **Hypertension** | **Hyperlipidemia** |
| fib | male | 17 | 0 | - | 4.21 | 42 | 65 | 1 | - | - | - |
| ca01 | male | 70 | 1144.41 | + | 2.96 | 37 | 65 | 3 | - | - | - |
| ca02 | male | 57 | 1901.39 | - | 4.06 | 35 | 62 | 3 | - | - | - |
| ca03 | male | 58 | 2726.96 | - | 4.5 | 54 | 63 | 4 | - | - | - |

Supplementary Table 2: Patients information for 7 samples of immunostaining

| **Patient** | **Sample Type** | **Sex** | **Age** |
| --- | --- | --- | --- |
| Patient a | CAVD | Male | 59 |
| Patient b | CAVD | Male | 65 |
| Patient c | CAVD | Female | 63 |
| Patient d | CAVD | Male | 49 |
| Patient e | CAVD | Male | 71 |
| Patient f | CAVD | Male | 58 |
| Patient g | CAVD | Female | 65 |

| Gene Symbol | Forward Primers | Reverse Primers | Product Length |
| --- | --- | --- | --- |
| GAPDH | CTGGGCTACACTGAGCACC | AAGTGGTCGTTGAGGGCAATG | 101 |
| ACTA2 | GTGTTGCCCCTGAAGAGCAT | GCTGGGACATTGAAAGTCTCA | 109 |
| BMP2 | ACCCGCTGTCTTCTAGCGT | TTTCAGGCCGAACATGCTGAG | 180 |
| S100A4 | GATGAGCAACTTGGACAGCAA | CTGGGCTGCTTATCTGGGAAG | 123 |
| RUNX2 | TGGTTACTGTCATGGCGGGTA | TCTCAGATCGTTGAACCTTGCTA | 101 |
| SPP1 | GAAGTTTCGCAGACCTGACAT | GTATGCACCATTCAACTCCTCG | 91 |
| ELN | GCAGGAGTTAAGCCCAAGG | TGTAGGGCAGTCCATAGCCA | 148 |
| MMP9 | AGACCTGGGCAGATTCCAAAC | CGGCAAGTCTTCCGAGTAGT | 94 |
| MMP2 | AAGCGGTCAGTGAGAAGGAAG | GGGGCCGTGTAGATAAACTCTAT | 136 |
| TIMP1 | ACCACCTTATACCAGCGTTATGA | GGTGTAGACGAACCGGATGTC | 96 |
| TIMP2 | AAGCGGTCAGTGAGAAGGAAG | GGGGCCGTGTAGATAAACTCTAT | 136 |
| AR | GACGACCAGATGGCTGTCATT | GGGCGAAGTAGAGCATCCT | 106 |
| FOXC1 | TGTTCGAGTCACAGAGGATCG | ACAGTCGTAGACGAAAGCTCC | 122 |
| NFIB | AAAAAGCATGAGAAGCGAATGTC | ACTCCTGGCGAATATCTTTGC | 136 |
| SOX9 | AGCGAACGCACATCAAGAC | CTGTAGGCGATCTGTTGGGG | 85 |

Supplementary Table 3: Information of primers used in RT-qPCR.
